# Mechanisms of Injury for Traumatic Brain Injury Among U.S. Military Service Members Before and During the COVID-19 Pandemic

**DOI:** 10.1093/milmed/usae492

**Published:** 2024-11-02

**Authors:** Tajrina Hai, Yll Agimi, Tesfaye Deressa, Olivia Haddad

**Affiliations:** Traumatic Brain Injury Center of Excellence, Silver Spring, MD 20910, USA; Compass Government Solutions, Annapolis, MD 21401, USA; Traumatic Brain Injury Center of Excellence, Silver Spring, MD 20910, USA; Traumatic Brain Injury Center of Excellence, Silver Spring, MD 20910, USA; Compass Government Solutions, Annapolis, MD 21401, USA; Traumatic Brain Injury Center of Excellence, Silver Spring, MD 20910, USA; Compass Government Solutions, Annapolis, MD 21401, USA

## Abstract

**Objective:**

To understand the mechanisms of injury and demographic risk factors associated with traumatic brain injury (TBI) patients among active and reserve service members in the U.S. Military before and during the COVID-10 pandemic.

**Methods:**

Active and reserve service members diagnosed with an incident TBI from January 2019 through September 2021 were selected. Traumatic brain injury patients diagnosed before March 1, 2020 were categorized as pre-COVID (PC), and patients diagnosed on or after March 1, 2020 were categorized as the intra-COVID (IC) group, aligning closely with the date when the World Health Organization officially proclaimed the pandemic. We determined the frequency of causes of injuries associated with TBI separate by sex, age, occupation, and TBI severity. In addition, we conducted multivariate logistic regression analyses to assess the demographic risk factors associated with TBI severity during the PC and IC eras.

**Results:**

Our cohort included 48,562 TBI patients: 22,819 (47.0%) diagnosed during the PC era and 25,743 (53.0%) diagnosed during the IC era. The major mechanisms of injury within our TBI cohort were being struck by/against objects, falls/slips/trips, and motor vehicle traffic accidents before and during the pandemic. The most common causes of TBI were not impacted by COVID, but motor vehicle accidents did increase during the IC era. The mechanisms of injury associated with TBI differed by TBI severity: being struck by or against an object caused more mild and moderate TBI; motor vehicle accidents caused more severe TBI; and firearms was a major cause of penetrating TBI. In addition, the percentage of severe TBI because of firearms rose sharply during the IC era. Further, women were more likely to be diagnosed with mild TBI compared to men.

**Conclusion:**

Military leaders should consider how different causes of injury are associated with differing TBI severities and how certain demographic groups were vulnerable to specific TBI severities when developing injury prevention programs.

## INTRODUCTION

Traumatic Brain Injury (TBI) can be caused through different mechanisms of injury ranging from everyday activities like playing contact sports, involvement in a car crash or falling and striking one’s head. Determining the cause of injury for TBI is critical for developing and evaluating policies to prevent, treat, and care for TBI patients. With nearly half a million active and reserve service members (SMs) diagnosed with TBI from 2000 to 2023,^1^ TBI has been considered a significant public health issue for the military. Caused by a “bump, blow, or jolt to the head or a penetrating head injury that disrupts the normal function of the brain,” TBI ranges from “mild” or a brief change in mental status or concussion to “severe,” an extended period of loss of consciousness or amnesia after the injury.^2^

Military SMs are at an additional risk of brain injury from blast exposures during combat or training exercises.^3^ The most recent study on causes of injury among SMs published a decade ago identified motor vehicle traffic accidents, falls, and being struck by or struck against an object were the most frequent causes of TBI among SMs.^4^ Recent epidemiological evidence of mechanisms of injury among SMs with TBI has been lacking, warranting a renewed study.

The COVID-19 pandemic inflicted widespread consequences on health systems around the world including TBI care within the U.S. Military. The first recorded COVID-19 cases in December 2019 rapidly spread worldwide with the World Health Organization declaring a pandemic in March 2020.^5^ To combat rising COVID-19 cases and prevent the overload of healthcare systems, governments instructed people to remain at home and limit face-to-face contact. The U.S. Military health facilities curtailed operations, trainings, and leisure activities and reduced and suspended services, impacting access to care and care-seeking behaviors.^6^ As COVID-19 infections receded, restrictions on health facilities were lifted and services were reinstated.

Among the civilian population, the predominant mechanisms of injury for TBI before the COVID-19 pandemic were road traffic accidents and falls.^7^ Lockdowns and road closures resulted in global reductions in road traffic levels and TBI associated with road traffic accidents decreased during the initial stages of the lockdown.^8,9^ However, during the latter stages of lockdown some studies showed that the rates of road traffic accidents increased to levels seen before the pandemic, possibly because of quieter, uncongested roads that encouraged careless driving and excessive use of speed.^10,11^

The aim of this study was to identify the leading mechanisms of injury of TBI and the demographic risk factors associated with TBI severity among SMs diagnosed before and during the COVID-19 pandemic.

## METHODS

### TBI Cohort

Service members who were on active duty or activated reserve/guard at the time of injury and had their first incident TBI between January 1, 2019 and September 30, 2021 were included in this study. Traumatic brain injury patients diagnosed before March 1, 2020 were categorized as pre-COVID; patients diagnosed on or after March 1, 2020 were categorized as the intra-COVID group, aligning closely with the date when the World Health Organization officially proclaimed the pandemic.^5^ We used the Armed Forces Health Surveillance Health Division’s case definition to identify the incident date of TBI as the first hospitalization or outpatient encounters that includes a qualifying TBI diagnosis code in any diagnostic position within the DoD’s medical record. The severity of each patient’s TBI diagnosis was determined using these definitions.^12^ Only one TBI-related medical encounter per SM was included for analysis. Records with only a history of TBI-related encounters, codes DOD0101-DOD0105, were excluded. This study was approved by the U.S. Army Public Health Review Board (DV-15-04).

### Data Sources

Five data systems in the Military Health System Data Repository that combined data from both hospitalizations and ambulatory care in military and civilian facilities were analyzed. The Comprehensive Ambulatory Patient Record captured ambulatory care and Standard Inpatient Data Record recorded inpatient health care data in military treatment facilities. In some military treatment facilities, where MHS GENESIS replaced the electronic medical record system, this data system was analyzed. The TRICARE Encounter Data-Institutional and TRICARE Encounter Data Non-Institutional captured purchased care data including ambulatory care, inpatient consultations, and the emergency department visits in civilian or Veteran’s administration facilities. These five data sources coded mechanisms of injury using the International Statistical Classification of Diseases and Related Health Problems, 10th revision, Clinical Modification System (ICD-10 CM).

### Mechanisms of Injury Encounter

We analyzed medical encounters 7 days before and 12 days after the initial TBI diagnosis date since providers may or may not code for the mechanism of injury at the incident TBI visit. The primary injury mechanism for each TBI patient was based on the medical encounter closest to the TBI diagnosis date. For TBI patients who had multiple causes of injury on the same encounter, all were included: this was deemed appropriate since providers often report multiple causes of injury to fully explain the TBI case.

### Mechanisms of Injury Categories

The mechanism of injury for TBI were ascertained from ICD-10 CM codes reported on TBI-related inpatient and outpatient encounters in military and civilian treatment facilities. We identified the injury category based on the methodology employed by the U.S. Army, a modified methodology based on the National Center for Health Statistics.^13,14^ For example, the code, W16011, describes a fall into swimming pool striking water surface causing drowning and submersion. The National Center for Health Statistics categorized this code as drowning; however, the U.S. Army categorized this code as fall/slip/trip since preventing the fall would prevent the drowning. The focus of the U.S. Army’s categories is for preventative planning purposes. Supplementary Appendix 1 lists the injury categories and the associated ICD-10 CM codes.

### Statistical Analyses

Descriptive statistics, including frequency distribution and chi-squared tests of significance, were used to determine if select demographic characteristics differed pre-COVID (PC) and intra-COVID (IC) among SMs diagnosed with TBI and SMs diagnosed with TBI with a mechanism of injury code. We determined the frequency and chi-squared statistics of causes of injuries by select demographic characteristics. Logistic regression models were constructed to test the adjusted associations of the demographic factors and causes of injury on TBI severity. Analyses were completed using SAS 9.4 (Cary, North Carolina) and graphics were produced using R software (R version 4.3.1, http://www.r-project.org).

## RESULTS

### Demographics of TBI Patients

We identified 48,562 TBI patients diagnosed from January 1, 2019 through September 30, 2021 (Supplementary Table S1). Of these, 22,819 (47.0%) TBI patients were diagnosed before March 1, 2020, during the pre-COVID era and 25,743 (53.0%) TBI patients were diagnosed during the COVID pandemic.

Characteristics of TBI patients were similar before and during the COVID pandemic as determined by the chi-squared statistic (*X*^2^  *P* > .05). The majority were male (81.5% vs. 80.3%; PC vs. IC, correspondingly), White (59.2% vs. 58.9%), served in the Army (58.3% vs. 58.2%), active duty (85.8% vs. 84.9%), and had mild TBI (84.8% vs. 83.2%). Less than half of TBI patients (46.7% vs. 47.7%) were 25 years old or younger. The vast majority of TBI patients had not deployed at the time of sustaining TBI (97.0% vs. 99.1%). Nearly three-quarters of TBI patients were first diagnosed in the outpatient clinics in military treatment facilities (74.8% vs. 74.0%) and one-fifth in outpatient civilian treatment facilities (20.1% vs. 21.0%). Over two-thirds of TBI patients were involved in repair/engineering, infantry/artillery/combat engineering, and communication/intelligence occupations.

### Demographics of TBI Patients with a Mechanism Cause of Injury

The demographic trends observed among TBI patients with a mechanism of injury code were similar to that of the overall cohort (Supplementary Table S1). Most were male (78.0% PC vs. 77.2% IC), White (57.1% PC vs. 56.6% IC), and served in the Army (55.6% PC vs. 58.8% IC). Slightly higher proportions of mild TBI patients had a cause of injury recorded in the PC era (79.6%) compared to the IC era (76.4%), and slightly higher proportion of moderate TBI patients had a mechanism of injury in the IC era (22.2%) vs. the PC era (18.8%). The majority of TBI patients with a mechanism of injury code were under 25 years old (61.6% PC vs. 59.6% IC). Over one-quarter of TBI patients in the repair/engineering occupation listed a cause of injury (25.9% PC vs. 25.7% IC). Most TBI patients with any mechanism of injury code were recorded in military outpatient facilities (71.7% PC vs. 70.0% IC).

### Mechanism of Injuries by Select Demographics

#### Sex and age

**Figure 1.**
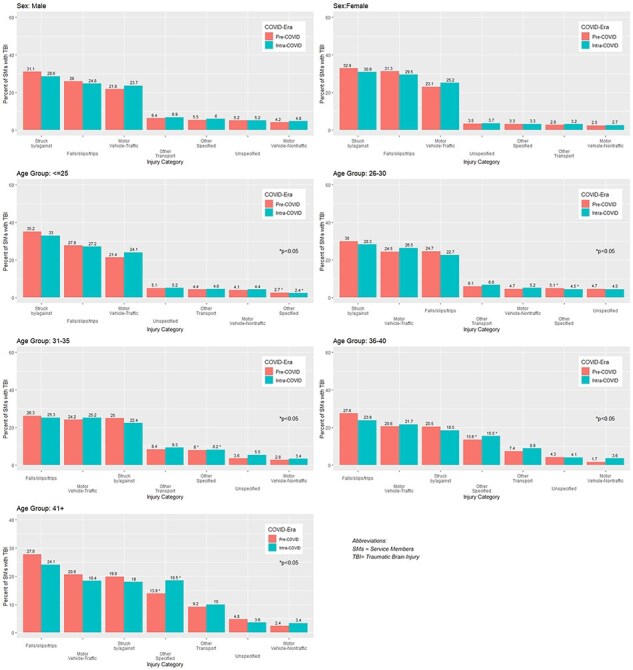
Select mechanisms of injury associated with TBI before (pre-COVID) and during (intra-COVID) the COVID-19 pandemic, by sex and age group.

The top causes of TBI were the same for males and females during the PC and IC eras: being struck by/against objects, falls/slips/trips, and motor vehicle traffic accidents (Figure 1). Being struck by/against objects and falls/slips/trips were higher in the PC era compared to the IC era. Motor vehicle traffic accidents were slightly higher during the IC era vs. the PC era for both males (23.7% IC vs. 21.6% PC) and females (25.2% IC vs. 23.1% PC). These mechanisms of injury did not differ significantly across gender during the PC and IC eras.

Across age groups, the top 3 mechanisms of TBI were being struck by/against objects, falls/slips/trips, and motor vehicle traffic accidents, before and during the COVID-19 pandemic. One-third of patients under 25 years had their TBI caused by being struck by/against objects (35.2% PC vs. 33% IC). Traumatic brain injury because of motor vehicle traffic accidents were slightly higher among SMs that were 26-30 years of age (26.5% IC vs. 24.5% PC) and 31-35 years of age (25.2% IC vs. 24.2% PC). Traumatic brain injury because of falls/slips/and trips were lower for all age groups during the IC era compared to PC era.

Across age group, other specified injury mechanisms for SMs were found to be statistically significant (*P* < .05) but differed by age category. Service members over the age of 31 years were more likely to have their TBI caused by other specified injuries during the IC era compared to the PC era. For SMs under the age of 30 years, specific injuries were lower during the IC era compared to the PC era.

#### Occupation and TBI severity

**Figure 2.**
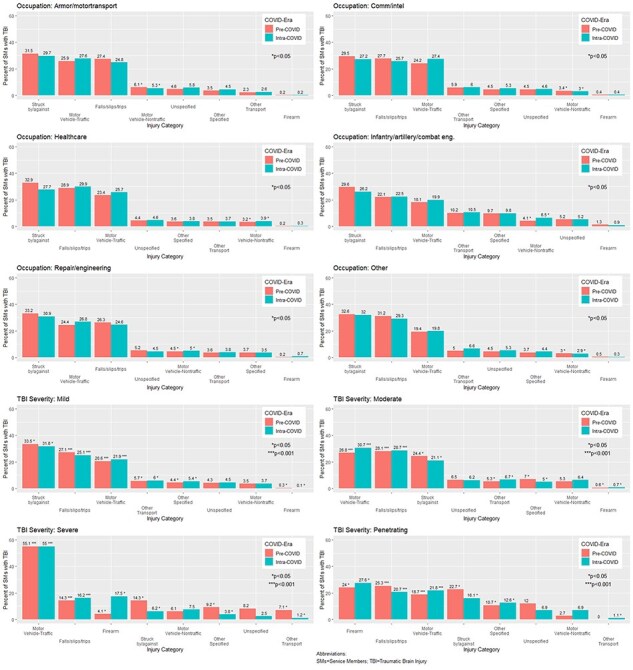
Select mechanisms of injury associated with TBI before (pre-COVID) and during (intra-COVID) the COVID-19 pandemic, by occupation and TBI severity.

Across occupations, the top 3 mechanisms of injury for TBI were motor traffic accidents, being struck by/against objects, and falls/slips/trips (Figure 2). Traumatic brain injury because of being struck by/against objects were slightly lower among TBI patients in armor/motor transport (31.5% PC vs. 29.7% IC), communication/intelligence (29.5% PC vs. 27.2% IC), health care (32.9% PC vs. 27.7% IC), infantry/artillery/combat engineering (29.6% PC vs. 26.2% IC), repair/engineering (33.2% PC vs. 30.9% IC), and other (32.6% PC vs. 32.0% IC) occupations during the pandemic: these results were not statistically significant across the time periods. For all occupations, TBI because of motor vehicle traffic accidents were slightly higher during the COVID pandemic than before the pandemic though the results were not statistically significant. Nontraffic-related motor vehicle injuries were higher for SMs in infantry/artillery/combat engineering (4.1% PC vs. 6.5% IC, *P*  < .05) and repair/engineering occupations were higher during the IC era (4.5% PC vs. 5.0% IC, *P* < .05).

The top mechanisms of injury differed by TBI severity. One-third of mild TBI occurred because of being struck by/against object (33.5% PC vs. 31.8% IC, *P*  < .05). Falls, slips, and trips caused fewer mild TBIs during the IC era (25.1%) compared to the PC era (27.1%) (*P*  < .001). Among moderate (26.8% PC vs. 30.7% IC, *P*  < .001) and severe TBI patients (55.1% PC vs. 55% IC, *P*  < .001), motor vehicle traffic accidents were the most likely cause of their TBI. In addition, causes of injury from nontraffic-related motor vehicle incidents were greater during the IC era vs. PC era (5.3% PC vs. 6.4% IC) for all severities. Firearms caused more severe (4.1% PC vs. 17.5% IC, *P*  < .001) and penetrating (24% PC vs. 27.6% IC, *P*  < .001) TBI during the pandemic than before. Pre-COVID, the top mechanism of injury among penetrating TBI patients was falls, slips, and trips (25.3% PC vs. 20.7% IC, *P* < .001).

#### Multivariate logistic regression of factors for TBI severity

Different demographics and mechanisms of injury were associated with different TBI severities (Supplementary Table S2). Women were more likely to be associated with mild TBI before (OR: 1.37, 95% CI: 1.20-1.57) and during the pandemic (OR: 1.65, 95% CI: 1.46-1.86). Men were more likely to be diagnosed with moderate, penetrating, and severe TBI since females were less likely to be diagnosed within these TBI severities during the PC and IC eras. Older age groups were more likely to be associated with moderate TBI during the IC era, with SMs in the 31 to 35 year-old (OR: 1.24, 95% CI: 1.07-1.45), 36 to 40 year-old (OR: 1.31, 95% CI: 1.10-1.56), and 41+ age groups (OR: 1.23, 95% CI: 1.02-1.47) being at higher risk.

Traumatic brain injury severity was diagnosed differently by clinical setting: more severe TBI was diagnosed outside of military outpatient settings. Moderate TBI patients were more likely to be diagnosed in civilian ambulatory (PC: OR: 3.44, 95% CI: 3.05-3.87; IC: OR: 3.07, 95% CI: 2.75-3.42), civilian hospital (PC: OR: 9.91, 95% CI: 8.08-12.16; IC: OR: 12.40, 95% CI: 10.19-15.10), and military hospitals (PC: OR: 5.09, 95% CI: 4.11-6.29; IC: OR: 6.50, 95% CI: 5.35-7.89) before and during the pandemic. Severe TBI patients were more likely to be diagnosed in civilian hospital settings (PC: OR: 2.16, 95% CI: 1.27-3.68; IC: OR: 3.18, 95% CI: 1.72-5.90). Penetrating TBI patients were more likely to be diagnosed within civilian ambulatory (PC: OR: 2.15, 95% CI: 1.22-3.80; IC: OR: 6.26, 95% CI: 3.49-11.22), civilian hospital (PC: OR: 9.60, 95% CI: 5.20-17.74; IC: OR: 16.82, 95% CI: 8.76-32.27), and military hospital settings (PC: OR: 4.46, 95% CI: 2.02-9.86; IC: OR: 8.18, 95% CI: 3.89-17.22).

Certain occupations, military services, active/guard status, and deployment status were more likely to be associated with specific TBI severities. Traumatic brain injury patients in the infantry/artillery/combat occupation were more likely to be diagnosed as moderate during COVID (OR: 1.16, 95% CI: 1.01-1.33). Service members serving in the Air Force were more likely to be diagnosed with mild TBI before (OR: 1.36, 95% CI: 1.17-1.58) and during the COVID pandemic (OR: 1.18, 95% CI: 1.02-1.35). Serving in the Guard (OR: 1.35, 95% CI: 1.12-1.64) and Reserve (OR: 1.25, 95% CI: 1.01-1.56) showed a modest increased risk in mild TBI and increased post-COVID (Guard: OR: 1.64, 95% CI: 1.38-1.95, Reserve: OR: 1.48, 95% CI: 1.21-1.82). Active duty SMs were more likely to be diagnosed with moderate TBI during the PC and IC eras since Guard (PC: OR: 0.73, 95% CI: 0.60-0.89; IC: OR: 0.59, 95% CI: 0.49-0.70) and Reserve (PC: OR: 0.73 95% CI: 0.59-0.92; IC: OR: 0.68, 95% CI: 0.55-0.84) were less likely to be diagnosed. During the pandemic, TBI patients who were deployed were more likely to be diagnosed with severe TBI (OR: 7.02, 95% CI: 2.45-20.14).

Specific mechanisms of injury were associated with different TBI severities. Being struck by or against an object was a cause of injury that was associated with mild TBI before (OR: 1.28, 95% CI: 1.11-1.47) and during (OR: 1.47, 95% CI: 1.29-1.68) the COVID-19 pandemic. Suffocation before (OR: 3.22, 95% CI: 1.44-7.22) and during the pandemic (OR: 3.93, 95% CI: 1.38-11.25) was associated with increased risk of moderate TBI. Firearms (PC: OR:12.91, 95% CI: 3.90-42.79; IC: OR: 48.24, 95% CI: 20.26-114.86), motor vehicle traffic accidents (PC: OR: 4.51, 95% CI: 2.48-8.22; IC: OR: 3.13, 95% CI: 1.66-5.90), and suffocation (PC: OR: 12.05, 95% CI: 2.34-61.93; IC: OR: 34.37, 95% CI: 7.44-158.71) were the mechanisms of injury associated with increased risk of being diagnosed with severe TBI during the PC and IC eras. Firearms was one of the mechanisms most likely associated with penetrating TBI patients before (OR: 41.32, 95% CI: 18.03-94.70) and during (OR: 46.18, 95% CI: 21.59-98.81) the COVID-19 pandemic.

## DISCUSSION

The major mechanisms of injury within our TBI cohort were being struck by/against objects, falls/slips/trips, and motor vehicle traffic accidents before and during the pandemic. A similar result was found among SMs with TBI from 2001 to 2011.^4^ Injury trends associated with TBI in SMs were similar to that observed in the civilian population. Among civilians, the two major leading mechanisms of injury for TBI were falls and motor vehicle crashes and occurred among the elderly (age 65 years and above) or young (under 25 years of age); men in the civilian population were more likely to be diagnosed with TBI.^7,15,16^ However, in our military cohort, younger TBI patients, or those under 25, were more likely to have their TBI caused by being struck by/against an object.

The common injury mechanisms for TBI were not impacted by COVID, but motor vehicle accidents did increase during the IC era. Traumatic brain injury because of motor vehicle traffic accidents was slightly higher during the era of COVID-19 compared to before and this aligned with research observed in the civilian population.^17–19^ Among civilians, the increase in crash fatalities was because of an increase in drivers’ risky behaviors including increased alcohol intake, speeding, failing to put on a seat belt, and failing to signal.^18,20^ A similar pattern was observed among the U.S. Army with increases in crash fatalities in 2020 associated with speeding, higher drugs or alcohol usage, and fewer people wearing seat belts.^21^

The most frequent mechanisms of injury differed by TBI severity. For mild TBI, being struck by/against an object was most prevalent mechanism of injury. Motor vehicle traffic accidents were among the most frequent causes of injury for severe and moderate TBI patients. Among penetrating TBI patients, one-quarter was because of firearms. The use of firearms sharply rose for severe and modestly increased for penetrating TBIs during the pandemic. Research among civilians showed an increase in TBI because of firearms as a result of higher anxiety and depression attributable to social isolation.^8,22,23^ Interestingly, these trends were not too dissimilar to the study conducted over a decade ago where TBIs treated in military hospital from 2000 to 2011 reported gun/explosive accidents as more likely to cause “severe” or “penetrating” TBI.^4^ In our study, more mild and moderate TBI patients had their TBI because of being struck by or against an object than mild and moderate TBI patients diagnosed a decade ago.^4^

In these analyses, women were likely to be diagnosed with mild TBI and men were more likely to be diagnosed with other severe forms of TBI—moderate, severe, and penetrating. Although research on TBI among women is limited, some evidence exists that women experience worse outcomes with mild TBI than men.^24^

The unique challenges that occurred during the pandemic could have hindered the ability of SMs to seek care for their TBI. Mental health issues including anxiety, depression, increased alcohol use, and suicidal ideation were prevalent among SMs during COVID.^23^ In addition, the military health system experienced delays in care, staffing shortages, material challenges, and difficulties in rapidly switching to telehealth when lockdowns occurred.^25^ Before the pandemic, SMs still faced many obstacles seeking care for TBI including beliefs that TBI does not require care, fear of jeopardizing career, and varying knowledge about TBI and its treatment.^26,27^ The combined impact of stigma associated with TBI, greater prevalence of mental health issues, and limited access to health care could have impacted SMs seeking care for TBI. As COVID-19 infections receded, restrictions on health facilities were lifted and health care services were reinstated, most TBI patients returned to outpatient facilities to be diagnosed.

### Limitations

Limitations exist with any study using electronic health records. The completeness of coding the mechanism of injury varied by clinical setting. Civilian clinical settings were more likely to code a mechanism of injury associated with TBI than military clinical settings. The more severe the TBI, the more likely a mechanism of injury was recorded (data not shown). Clinical setting played a role in TBI severity with more severe TBI being diagnosed outside of military ambulatory settings. It is plausible that the mechanisms of injury observed in this study are biased toward more severe TBI and TBI patients treated in civilian clinical settings.

Furthermore, variations in coding protocol and practices by site and provider can bias the data. The Military Health System Data Repository, used as the source of data for this study, is a large health care dataset used not only to capture diagnoses but also health care utilization and billing. The quality of coding varies across hospitals and staff. When a patient is seen repeatedly in the same facility over months or years, clinical problems may be cut and pasted from one encounter to the next, regardless of whether the health problem is current. A diagnosis that should be coded as “history of” an injury can be coded as if the injury were part of the presenting problem list.

Regarding injury surveillance, the accuracy and consistency of the mechanism of injury coding can be impacted if a coding specialist or provider enters the data into the electronic health record system in civilian emergency departments. A previous study showed that providers were shown to have higher completeness of coding compared to professional coders when they were doing their own coding, and these coding practices could vary from coding specialists. In addition, users of the electronic health record system may perpetuate the injury diagnoses when documenting the patients’ encounter because of the features of the system even when the injury is no longer being treated.^28^ These coding variation practices could affect the precision of the mechanism of injury coding among our TBI cohort seen within the civilian ambulatory or hospital settings.

Our study may have underestimated the mechanisms of injuries associated with TBI during the IC era because patients were unable to seek care. Health care facilities implemented strict guidelines during the pandemic, particularly in the beginning. Service members who sustained a TBI may have been hindered in seeking or accessing care. An expanded analyses looking at mechanisms of injury before and after the pandemic would provide more reliable data when health care facilities were not restricting patients that sought care.

An advantage of these analyses compared to the previous study looking at mechanisms of TBI from 2001 to 2011 is that this study used ICD-10 CM codes. Compared to the previous ICD-9 system, ICD-10 CM changed to an alphanumeric system which expanded the number of codes and increased the definitional precision of mechanisms of injury. The causes of injury chapter were grouped into blocks of related conditions, at the highest level according to the intent behind the incident (e.g., accidental, intentional self-harm, assault). Place of occurrence codes were expanded and applied to a wider range of codes. Activity codes were introduced. Transport accidents in ICD-10 were grouped into smaller blocks according to the characteristics of the injured person (pedestrian, motorcycle rider, etc.), whereas, in ICD-9, transport accidents were grouped by vehicle type. Coding rules for the selection of underlying causes of death were modified, potentially affecting assignment of injury codes.^29^

### Implications for Future Research

Because the mechanisms of injury associated with TBI in the military aligned with what is observed within the civilian population before and during the COVID pandemic, military leaders can look to nonmilitary injury prevention programs to adopt similar policies for TBI prevention. Further, military leaders should consider how different mechanisms of injury are associated with differing TBI severities and how certain demographic groups were vulnerable to specific TBI severities when developing injury prevention programs. Creating programs that reduce causes of injury associated with TBI could potentially reduce the burden of TBI observed among SMs.

## CONCLUSION

Characterizing the mechanisms of injury associated with TBI can help military leaders and policymakers develop injury prevention and safety programs that may assist in preventing TBI. Knowledge of common mechanisms of injury of TBI and their association with specific TBI severities can support clinicians in triaging their TBI patients more effectively and identify the cause of injury. Further, because the mechanisms of injury associated with TBI among SMs and civilians were similar, the military could look to adopt practices of civilian injury prevention programs to reduce TBI. However, how those injuries occur among SMs vs. civilians may be different. Therefore, military and policy leaders should consider how to develop injury prevention programs that account for military training and lifestyle.

## Supplementary Material

usae492_Supp

## Data Availability

The data that support the findings of this study are available upon request from the corresponding author.

## INSTITUTIONAL CLEARANCE

Institutional Clearance Approved.
