# Supplementary material for: Mechanisms of Injury for Traumatic Brain Injury Among U.S. Military Service Members Before and During the COVID-19 Pandemic
Source: Mil Med. 2024 Nov 2;190(3-4):e830–7. doi: 10.1093/milmed/usae492 (PMC11878788; doi:10.1093/milmed/usae492)
Supplement: usae492_Supp [file usae492_supp.zip › SupplementalMaterial_TableS2_01July2024 (002).docx]

Supplemental Table S2. US Military Service Members (SMs) demographic characteristics associated with Traumatic Brain Injury (TBI) Severity, Pre-COVID (PC) and Intra-COVID (IC) eras

|  | **TBI Severity** | | | | | | | |
| --- | --- | --- | --- | --- | --- | --- | --- | --- |
| **Characteristic** | **Mild** | | **Moderate** | | **Severe** | | **Penetrating** | |
|  | **PC^₸^** | **IC^₭^** | **PC^₸^** | **IC^₭^** | **PC^₸^** | **IC^₭^** | **PC^₸^** | **IC^₭^** |
| **Sex** | | | | | | | | |
| Male | Ref | Ref | Ref | Ref | Ref | Ref | Ref | Ref |
| Female | 1.37  (1.20 - 1.57)*** | 1.65  (1.46 - 1.86)*** | 0.81  (0.71 - 0.93)* | 0.64  (0.57 - 0.73)*** | 0.17  (0.07 - 0.42)*** | 0.16  (0.06 - 0.44)*** | 0.22 (0.08 - 0.64)* | 0.65 (0.34 - 1.23) |
| **Age Group** | | | | | | | | |
| <=25 | Ref | Ref | Ref | Ref | Ref | Ref | Ref | Ref |
| 26-30 | 0.94  (0.82 - 1.08) | 0.99  (0.87 - 1.12) | 0.99  (0.86 - 1.14) | 1.00  (0.89-1.14) | 1.22  (0.76 - 1.96) | 1.44  (0.84 - 2.44) | 2.20  (1.32 - 3.67)* | 0.98  (0.55-1.76) |
| 31-35 | 0.86  (0.72 - 1.02) | 0.79  (0.68 - 0.92)* | 1.19  (1.00 - 1.41) | 1.24  (1.07-1.45)* | 1.36  (0.75 - 2.48) | 0.83  (0.38 - 1.83) | 0.40  (0.12 - 1.35) | 1.60  (0.89-2.88) |
| 36-40 | 1.10  (0.89 - 1.36) | 0.78  (0.65 - 0.93)* | 0.93  (0.75 - 1.15) | 1.31  (1.10-1.56)* | 0.48  (0.17 - 1.34) | 0.72  (0.27 - 1.91) | 1.37  (0.60 - 3.11) | 0.95  (0.42-2.13) |
| 41+ | 0.89  (0.73 - 1.09) | 0.82  (0.69 - 0.99)* | 1.13  (0.92 - 1.39) | 1.23  (1.02-1.47)* | 1.26  (0.64 - 2.49) | 1.88  (0.88 - 4.04) | 0.74  (0.25 - 2.16) | 0.47  (0.16-1.39) |
| **Ethnicity** | | | | | | | | |
| White non-Hispanic | Ref | Ref | Ref | Ref | Ref | Ref | Ref | Ref |
| American Indian | 1.21  (0.67 - 2.16) | 0.61  (0.39 - 0.96)* | 0.75  (0.41 - 1.37) | 1.60  (1.02 - 2.51)* | 2.55  (0.73 - 8.87) | NR | NR | 2.48  (0.56 - 11.03) |
| Asian/Pacific Islander | 1.18  (0.88 - 1.58) | 1.15  (0.90 - 1.46) | 0.93  (0.69 - 1.25) | 0.85  (0.67 - 1.09) | NR | 0.60  (0.14 - 2.59) | 1.12  (0.34 - 3.73) | 1.88  (0.78 - 4.53) |
| Black non-Hispanic | 1.13  (0.98 - 1.30) | 1.03  (0.91 - 1.17) | 0.92  (0.80 - 1.07) | 0.98  (0.86 - 1.11) | 0.82  (0.49 - 1.36) | 0.71  (0.38 - 1.35) | 0.42  (0.19 - 0.95)* | 1.16  (0.63 - 2.13) |
| Hispanic | 1.02  (0.89 - 1.16) | 1.13  (0.99 - 1.28) | 1.02  (0.89 - 1.16) | 0.87  (0.77 - 0.99)* | 0.77  (0.46 - 1.30) | 1.37  (0.80 - 2.34) | 0.78  (0.42 - 1.43) | 1.13  (0.65 - 1.98) |
| Other | 1.41  (1.09 - 1.84)* | 1.02  (0.80 - 1.29) | 0.76  (0.58 - 0.99)* | 0.98  (0.78 - 1.25) | 0.53  (0.16 - 1.71) | 1.32  (0.53 - 3.29) | 0.40  (0.09 - 1.72) | 0.57  (0.16 - 2.06) |
| **Clinical Setting** | | | | | | | | |
| Military-ambulatory | Ref | Ref | Ref | Ref | Ref | Ref | Ref | Ref |
| Military-hospital | 0.20  (0.16 - 0.24)*** | 0.14  (0.12 - 0.18)*** | 5.09  (4.11 - 6.29)*** | 6.50  (5.35 - 7.89)*** | 0.99  (0.42 - 2.33) | 0.97  (0.34 - 2.79) | 4.46  (2.02 - 9.86)*** | 8.18  (3.89 - 17.22)*** |
| Civilian ambulatory | 0.31  (0.28 - 0.35)*** | 0.31  (0.28 - 0.35)*** | 3.44  (3.05 - 3.87)*** | 3.07  (2.75 - 3.42)*** | 0.47  (0.26 - 0.84)* | 1.53  (0.90 - 2.60) | 2.15  (1.22 - 3.80)* | 6.26  (3.49 - 11.22)*** |
| Civilian- hospital | 0.08  (0.07 - 0.10)*** | 0.06  (0.05 - 0.07)*** | 9.91  (8.08 - 12.16)*** | 12.40  (10.19 - 15.10)*** | 2.16  (1.27 - 3.68)* | 3.18  (1.72 - 5.90)*** | 9.60  (5.20 - 17.74)*** | 16.82  (8.76 - 32.27)*** |
| Combat theater | NR | 0 | NR | 0 | 0 | 0 | NR | 0 |
| **Occupation Group** | | | | | | | | |
| Armor/transport | 0.93  (0.72 - 1.21) | 0.96  (0.76 - 1.22) | 1.09  (0.83 - 1.43) | 0.94  (0.73 - 1.20) | 0.56  (0.17 - 1.84) | 3.23  (1.60 - 6.54)* | 1.45  (0.51 - 4.16) | 0.90  (0.30 - 2.73) |
| Comm/Intel | 1.01  (0.88 - 1.17) | 1.04  (0.91 - 1.19) | 1.01  (0.87 - 1.17) | 0.99  (0.86 - 1.13) | 0.85  (0.49 - 1.46) | 0.65  (0.35 - 1.23) | 0.86  (0.44 - 1.70) | 0.90  (0.48 - 1.69) |
| Healthcare | 0.99  (0.81 - 1.21) | 0.94  (0.78 - 1.13) | 1.03  (0.84 - 1.27) | 1.08  (0.89 - 1.30) | 1.15  (0.57 - 2.32) | 0.41  (0.12 - 1.38) | 0.61  (0.20 - 1.80) | 1.48  (0.65 - 3.36) |
| Infantry/artillery/combat | 1.08  (0.93 - 1.26) | 0.88  (0.77 - 1.01) | 0.96  (0.82 - 1.12) | 1.16  (1.01 - 1.33)* | 0.68  (0.40 - 1.18) | 0.58  (0.30 - 1.15) | 0.96  (0.50 - 1.84) | 1.13  (0.62 - 2.07) |
| Other | 1.05  (0.91 - 1.21) | 0.99  (0.86 - 1.13) | 0.96  (0.82 - 1.11) | 1.05  (0.92 - 1.20) | 1.07  (0.64 - 1.79) | 0.61  (0.32 - 1.17) | 0.75  (0.38 - 1.47) | 0.85  (0.45 - 1.63) |
| Repair/engineering | Ref | Ref | Ref | Ref | Ref | Ref | Ref | Ref |
| **Service Branch** | | | | | | | | |
| Army | Ref | Ref | Ref | Ref | Ref | Ref | Ref | Ref |
| Air Force | 1.36  (1.17 - 1.58)*** | 1.18  (1.02 - 1.35)* | 0.74  (0.64 - 0.87)*** | 0.84  (0.73 - 0.96)* | 0.56  (0.30 - 1.04) | 1.76  (0.99 - 3.14) | 1.04  (0.52 - 2.08) | 0.82  (0.40 - 1.68) |
| Navy | 1.00  (0.86 - 1.17) | 0.97  (0.84 - 1.12) | 0.96  (0.82 - 1.12) | 0.98  (0.85 - 1.13) | 1.17  (0.69 - 1.98) | 1.39  (0.73 - 2.63) | 1.84  (1.00 - 3.37)* | 1.66  (0.91 - 3.02) |
| Marines | 0.99  (0.85 - 1.15) | 0.87  (0.76 - 1.01) | 1.02  (0.87 - 1.20) | 1.10  (0.95 - 1.27) | 0.90  (0.51 - 1.58) | 1.03  (0.52 - 2.06) | 1.08  (0.55 - 2.11) | 1.89  (1.05 - 3.40)* |
| **Component** | | | | | | | | |
| Active Duty | Ref | Ref | Ref | Ref | Ref | Ref | Ref | Ref |
| Guard | 1.35  (1.12 - 1.64)* | 1.64  (1.38 - 1.95)*** | 0.73  (0.60 - 0.89)* | 0.59  (0.49 - 0.70)*** | 1.20  (0.60 - 2.39) | 1.52  (0.81 - 2.89) | 0.82  (0.34 - 1.98) | 1.13  (0.56 - 2.29) |
| Reserve | 1.25  (1.01 - 1.56)* | 1.48  (1.21 - 1.82)*** | 0.73  (0.59 - 0.92)* | 0.68  (0.55 - 0.84)*** | 0.73  (0.29 - 1.85) | 0.81  (0.32 - 2.03) | 2.92  (1.51 - 5.65)* | 1.00  (0.44 - 2.31) |
| **Deployment Status** | | | | | | | | |
| Non-Deployed | Ref | Ref | Ref | Ref | Ref | Ref | Ref | Ref |
| Deployed | 0.52  (0.36 - 0.75)*** | 0.68  (0.42 - 1.09) | 2.16  (1.50 - 3.13)*** | 1.12  (0.68 - 1.84) | 0.68  (0.16 - 2.95) | 7.02  (2.45 - 20.14)*** | 0.40  (0.08 - 2.15) | 2.25  (0.57 - 8.89) |
| **Injury Category** | | | | | | | | |
| Falls/slips/trips | Ref | Ref | Ref | Ref | Ref | Ref | Ref | Ref |
| Cut/Pierce | 1.85  (0.68 - 5.04) | 1.25  (0.67 - 2.34) | 0.46  (0.16 - 1.37) | 0.64  (0.33 - 1.24) | NR | NR | 3.23  (0.38 - 27.28) | 7.36  (1.94 - 27.90)* |
| Drowning/  Submersion | 0.34  (0.02 - 6.10) | 0.49  (0.08 - 3.04) | 3.39  (0.18 - 63.57) | 2.15  (0.35 - 13.24) | NR | NR | NR | NR |
| Fire/Burn | NR | NR | NR | NR | NR | NR | NR | NR |
| Firearm | 0.30  (0.17 - 0.56)*** | 0.09  (0.04 - 0.19)*** | 0.48  (0.24 - 0.95)* | 0.39  (0.21 - 0.72)* | 12.91  (3.90 - 42.79)*** | 48.24  (20.26 - 114.86)*** | 41.32  (18.03 - 94.70)*** | 46.18  (21.59 - 98.81)*** |
| Machinery | 2.18  (0.48 - 9.83) | 1.07  (0.45 - 2.57) | 0.50  (0.11 - 2.24) | 0.99  (0.42 - 2.36) | NR | NR | NR | NR |
| Motor Vehicle-Nontraffic | 0.76  (0.59 - 0.98)* | 0.75  (0.60 - 0.94)* | 1.30  (1.00 - 1.69)* | 1.27  (1.02 - 1.58)* | 2.55  (0.97 - 6.72) | 2.14  (0.80 - 5.76) | 0.50  (0.11 - 2.20) | 1.50  (0.58 - 3.88) |
| Motor Vehicle-Traffic | 0.97  (0.84 - 1.12) | 1.00  (0.88 - 1.14) | 0.94  (0.81 - 1.09) | 0.96  (0.84 - 1.09) | 4.51  (2.48 - 8.22)*** | 3.13  (1.66 - 5.90)*** | 0.60  (0.29 - 1.23) | 0.80  (0.41 - 1.54) |
| Natural/  Environmental | 1.31  (0.66 - 2.58) | 1.31  (0.70 - 2.48) | 0.71  (0.35 - 1.45) | 0.81  (0.44 - 1.52) | NR | NR | 2.81  (0.36 - 21.95) | NR |
| Other Land Transport | 0.95  (0.67 - 1.34) | 0.78  (0.57 - 1.05) | 0.91  (0.64 - 1.30) | 1.17  (0.86 - 1.59) | 5.75  (2.16 - 15.31)*** | 4.23  (1.45 - 12.32)* | 1.01  (0.22 - 4.54) | 1.61  (0.46-5.66) |
| Other Specified | 0.67  (0.53 - 0.84)*** | 1.22  (0.98 - 1.53) | 1.35  (1.07 - 1.72)* | 0.74  (0.59 - 0.94)* | 2.94  (1.19 - 7.25)* | 1.08  (0.30 - 3.93) | 2.32  (0.97 - 5.55) | 3.70  (1.68-8.15)* |
| Other Transport | 0.92  (0.72 - 1.18) | 0.85  (0.69 - 1.05) | 1.09  (0.85 - 1.40) | 1.19  (0.97 - 1.46) | 1.95  (0.77 - 4.95) | 0.40  (0.05 - 3.09) | NR | 0.50  (0.06-3.81) |
| Overexertion | 1.43  (0.66 - 3.09) | 1.91  (0.96 - 3.80) | 0.76  (0.35 - 1.64) | 0.56  (0.28 - 1.11) | NR | NR | NR | NR |
| Pedal cyclist, other | 0.76  (0.47 - 1.24) | 1.43  (0.95 - 2.13) | 1.46  (0.90 - 2.38) | 0.71  (0.48 - 1.07) | NR | NR | NR | 1.64  (0.37-7.38) |
| Pedestrian, other | 0.32  (0.13 - 0.78)* | 0.59  (0.25 - 1.38) | 2.92  (1.21 - 7.04)* | 1.49  (0.63 - 3.52) | 5.20  (0.64 - 42.26) | NR | NR | 5.51  (0.67-45.09) |
| Poisoning | 0.76  (0.42 - 1.36) | 1.94  (0.94 - 4.03) | 1.48  (0.83 - 2.65) | 0.53  (0.25 - 1.08) | NR | 5.75  (0.69 - 47.64) | NR | NR |
| Struck by/against | 1.28  (1.11 - 1.47)*** | 1.47  (1.29 - 1.68)*** | 0.78  (0.68 - 0.90)*** | 0.69  (0.60 - 0.78)*** | 0.85  (0.40 - 1.80) | 0.37  (0.13 - 1.05) | 0.93  (0.48 - 1.82) | 1.00  (0.49 - 2.04) |
| Suffocation | 0.16  (0.06 - 0.40)*** | 0.10  (0.03 - 0.37)*** | 3.22  (1.44 - 7.22)* | 3.93  (1.38 - 11.25)* | 12.05  (2.34 - 61.93)* | 34.37  (7.44 - 158.71)*** | 5.89  (1.18 - 29.43)* | NR |
| Unspecified | 0.88  (0.70 - 1.11) | 1.04  (0.84 - 1.29) | 1.01  (0.80 - 1.28) | 0.96  (0.77 - 1.19) | 3.12  (1.29 - 7.53)* | 0.74  (0.16 - 3.28) | 1.84  (0.81 - 4.23) | 1.30  (0.51-3.32) |
| Abbreviations*:* Ref, Reference Group; NR, Not Reported | | | | | | | | |
| Note: Data is presented as the odds ratio estimate on top and the associated 95% confidence interval in parenthesis below | | | | | | | | |
| ^₸^ The Pre-COVID (PC) era is from January 1, 2019 through February 29, 2020 | | | | | | | | |
| ^₭^ The Intra-COVID (IC) era is from March 1, 2020 through September 30, 2021 | | | | | | | | |
| * P-value <0.05 | | | | | | | | |
| ***P-value <0.001 | | | | | | | | |
