# Supplementary material for: Mechanisms of Injury for Traumatic Brain Injury Among U.S. Military Service Members Before and During the COVID-19 Pandemic
Source: Mil Med. 2024 Nov 2;190(3-4):e830–7. doi: 10.1093/milmed/usae492 (PMC11878788; doi:10.1093/milmed/usae492)
Supplement: usae492_Supp [file usae492_supp.zip › SupplmentalMaterial_TableS1_01July2024_TD_edits (002).docx]

Supplemental Table S1. Demographics of US Military Service Members (SMs) diagnosed with Traumatic Brain Injury (TBI) and SMs with a TBI and Mechanism of Injury Code

|  | **TBI Cohort** | | | | **TBI Cohort with a mechanism of injury code** | | | |
| --- | --- | --- | --- | --- | --- | --- | --- | --- |
|  | **PC^₸^** | **IC^₭^** | **Total** | **Mantal Haenszel Chi Square** | **PC^₸^** | **IC^₭^** | **Total** | **Mantal Haenszel Chi Square** |
|  | **N(%)** | **N(%)** | **N(%)** |  | **N(%)** | **N(%)** | **N(%)** |  |
| **Characteristic** | **N= 22,819** | **N= 25,743** | **N= 48,562** |  | **N=10,313** | **N=11,535** | **N=21,848** |  |
| Sex |  |  |  |  |  |  |  |  |
| Male | 18,585 (81.5) | 20,671 (80.3) | 39,256 (80.8) |  | 8,043 (78.0) | 8,903 (77.2) | 16,946 (77.6) |  |
| Female | 4,212 (18.5) | 5,056 (19.6) | 9,268 (19.1) |  | 2,259 (21.9) | 2,625 (22.8) | 4,884 (22.4) |  |
| Missing | 22 (0.1) | 16 (0.1) | 38 (0.1) | 0.002 | 11 (0.1) | 07 (0.1) | 18 (0.1) | 0.1812 |
| Age Group |  |  |  |  |  |  |  |  |
| <=25 | 10,651 (46.7) | 12,267 (47.7) | 22,918 (47.2) |  | 6,350 (61.6) | 6,879 (59.6) | 13,229 (60.6) |  |
| 26-30 | 3,777 (16.6) | 4,168 (16.2) | 7,945 (16.4) |  | 1,698 (16.5) | 1,944 (16.9) | 3,642 (16.7) |  |
| 31-35 | 2,693 (11.8) | 2,890 (11.2) | 5,583 (11.5) |  | 950 (9.2) | 1,055 (9.2) | 2,005 (9.2) |  |
| 36-40 | 2,651 (11.6) | 2,952 (11.5) | 5,603 (11.5) |  | 645 (6.3) | 807 (7.0) | 1,452 (6.7) |  |
| 41+ | 3,047 (13.4) | 3,466 (13.5) | 6,513 (13.4) | 0.002 | 670 (6.5) | 850 (7.4) | 1,520 (7.0) | 0.0843 |
| Ethnicity |  |  |  |  |  |  |  |  |
| White non-Hispanic | 13,504 (59.2) | 15,155 (58.9) | 28,659 (59.0) |  | 5,889 (57.1) | 6,530 (56.6) | 12,419 (56.8) |  |
| American Indian/Alaskan Native | 197 (0.9) | 211 (0.8) | 408 (0.8) |  | 80 (0.8) | 90 (0.8) | 170 (0.8) |  |
| Asian/Pacific Islander | 711 (3.1) | 940 (3.7) | 1,651 (3.4) |  | 326 (3.2) | 434 (3.8) | 760 (3.5) |  |
| Black non-Hispanic | 3,701 (16.2) | 4,097 (15.9) | 7,798 (16.1) |  | 1,763 (17.1) | 2,027 (17.6) | 3,790 (17.4) |  |
| Hispanic | 3,631 (15.9) | 4,176 (16.2) | 7,807 (16.1) |  | 1,789 (17.4) | 1,988 (17.2) | 3,777 (17.3) |  |
| Other | 1,075 (4.7) | 1,164 (4.5) | 2,239 (4.6) | 0.2413 | 466 (4.5) | 466 (4.0) | 932 (4.3) | 0.0882 |
| Clinical Setting |  |  |  |  |  |  |  |  |
| Military-ambulatory | 17,071 (74.8) | 19,059 (74.0) | 36,130 (74.4) |  | 7,396 (71.7) | 8,069 (70.0) | 15,465 (70.8) |  |
| Military-hospital | 562 (2.5) | 594 (2.3) | 1,156 (2.4) |  | 334 (3.2) | 394 (3.4) | 728 (3.3) |  |
| Civilian-ambulatory | 4,594 (20.1) | 5,394 (21.0) | 9,988 (20.6) |  | 2,178 (21.1) | 2,581 (22.4) | 4,759 (21.8) |  |
| Civilian-hospital | 588 (2.6) | 696 (2.7) | 1,284 (2.6) |  | 404 (3.9) | 491 (4.3) | 895 (4.1) |  |
| Combat theater | 4 (<0.01) | 0 (0.0) | 0 (0.0) | <0.001 | 1 (<0.01) | 0 (0.0) | 0 (0.0) | 0.0216 |
| Occupation |  |  |  |  |  |  |  |  |
| Armor/motortransport | 766 (3.4) | 944 (3.7) | 1,710 (3.5) |  | 394 (3.8) | 468 (4.1) | 862 (4.0) |  |
| Comm/intel | 5,082 (22.3) | 5,539 (21.5) | 10,621 (21.9) |  | 2,198 (21.3) | 2,466 (21.4) | 4,664 (21.4) |  |
| Healthcare | 1,910 (8.4) | 2,049 (8.0) | 3,959 (8.2) |  | 904 (8.8) | 929 (8.1) | 1,833 (8.4) |  |
| Infantry/artillery/combat eng. | 5,121 (22.4) | 5,929 (23.0) | 11,050 (22.8) |  | 1,941 (18.8) | 2,257 (19.6) | 4,198 (19.2) |  |
| Other | 4,625 (20.3) | 5,504 (21.4) | 10,129 (20.9) |  | 2,207 (21.4) | 2,446 (21.2) | 4,653 (21.3) |  |
| Repair/engineering | 5,315 (23.3) | 5,778 (22.4) | 11,093 (22.8) | 0.7722 | 2,669 (25.9) | 2,969 (25.7) | 5,638 (25.8) | 0.7784 |
| Service Branch |  |  |  |  |  |  |  |  |
| Army | 13,299 (58.3) | 14,978 (58.2) | 28,277 (58.2) |  | 5,738 (55.6) | 6,781 (58.8) | 12,519 (57.3) |  |
| Air Force | 3,333 (14.6) | 3,542 (13.8) | 6,875 (14.2) |  | 1,842 (17.9) | 1,766 (15.3) | 3,608 (16.5) |  |
| Navy | 3,014 (13.2) | 3,515 (13.7) | 6,529 (13.4) |  | 1,381 (13.4) | 1,574 (13.7) | 2,955 (13.5) |  |
| Marines | 3,173 (13.9) | 3,708 (14.4) | 6,881 (14.2) | 0.8084 | 1,352 (13.1) | 1,414 (12.3) | 2,766 (12.7) | <0.001 |
| Component |  |  |  |  |  |  |  |  |
| Active | 19,576 (85.8) | 21,846 (84.9) | 41,422 (85.3) |  | 8,974 (87.0) | 9,938 (86.2) | 18,912 (86.6) |  |
| Guard | 1,901 (8.3) | 2,370 (9.2) | 4,271 (8.8) |  | 789 (7.7) | 996 (8.6) | 1,785 (8.2) |  |
| Reserve | 1,342 (5.9) | 1,527 (5.9) | 2,869 (5.9) | 0.043 | 550 (5.3) | 601 (5.2) | 1,151 (5.3) | 0.2829 |
| Deployment Status |  |  |  |  |  |  |  |  |
| Non-deployed | 22,125 (97.0) | 25,503 (99.1) | 47,628 (98.1) | <0.0001 | 10,166 (98.6) | 11,448 (99.3) | 21,614 (98.9) |  |
| Deployed | 694 (3.0) | 240 (0.9) | 934 (1.9) |  | 147 (1.4) | 87 (0.8) | 234 (1.1) | <0.0001 |
| Severity |  |  |  |  |  |  |  |  |
| Mild | 19,358 (84.8) | 21,422 (83d.2) | 40,780 (84.0) |  | 8,206 (79.6) | 8,811 (76.4) | 17,017 (77.9) |  |
| Moderate | 3,224 (14.1) | 4,093 (15.9) | 7,317 (15.1) |  | 1,934 (18.8) | 2,557 (22.2) | 4,491 (20.6) |  |
| Penetrating | 96 (0.4) | 110 (0.4) | 206 (0.4) |  | 75 (0.7) | 87 (0.8) | 162 (0.7) |  |
| Severe | 141 (0.6) | 118 (0.5) | 259 (0.5) | 0.0008 | 98 (1.0) | 80 (0.7) | 178 (0.8) | <0.0001 |
| Death |  |  |  |  |  |  |  |  |
| No | 22,745 (99.7) | 25,632 (99.6) | 48,377 (99.6) |  | 10,258 (99.5) | 11,464 (99.4) | 21,722 (99.4) |  |
| Yes | 74 (0.3) | 111 (0.4) | 185 (0.4) | 0.0563 | 55 (0.5) | 71 (0.6) | 126 (0.6) | 0.4231 |
| ^₸^ The Pre-COVID (PC) era is from January 1, 2019 through February 29, 2020 | | | | | | | | |
| ^₭^ The Intra-COVID (IC) era is from March 1, 2020 through September 30, 2021 | | | | | | | | |
