# Supplementary material for: Mechanisms of Injury for Traumatic Brain Injury Among U.S. Military Service Members Before and During the COVID-19 Pandemic
Source: Mil Med. 2024 Nov 2;190(3-4):e830–7. doi: 10.1093/milmed/usae492 (PMC11878788; doi:10.1093/milmed/usae492)
Supplement: usae492_Supp [file usae492_supp.zip › Appendix_v2.docx]

**Appendix 1. International Classification of Diseases, 10th edition, Clinical Modification (ICD-10 CM) Codes Associated with select mechanisms of injury for Traumatic Brain Injury (TBI)**

| **Injury Category** | **ICD-10-CM** |
| --- | --- |
| Cut/Pierce | W25.xxx, W26.0xx, W26.1xx, W26.2xx, W26.8xx, W26.9xx, W27.0xx, W27.1xx, W27.2xx, W27.3xx, W27.4xx, W27.5xx, W27.8xx, W28.xxx, W29.0xx, W29.1xx, W29.2xx, W29.3xx, W29.4xx, W29.8xx, W45.0xx, W45.1xx, W45.2xx, W45.8xx, W46.0xx, W46.1xx, X78.0xx, X78.1xx, X78.2xx, X78.8xx, X78.9xx, X99.0xx, X99.1xx, X99.2xx, X99.8xx, X99.9xx, Y28.0xx, Y28.1xx, Y28.2xx, Y28.8xx, Y28.9xx, Y35.401, Y35.402, Y35.403, Y35.409, Y35.411, Y35.412, Y35.413, Y35.419, Y35.491, Y35.492, Y35.493, Y35.499 |
| Drowning/Submersion | T75.1xx, W65.xxx, W67.xxx, W69.xxx, W73.xxx, W74.xxx, X71.0xx, X71.1xx, X71.2xx, X71.3xx, X71.8xx, X71.9xx, X92.0xx, X92.1xx, X92.2xx, X92.3xx, X92.8xx, X92.9xx, Y21.0xx, Y21.1xx, Y21.2xx, Y21.3xx, Y21.4xx, Y21.8xx, Y21.9xx |
| Falls/slips/trips | V00.111, V00.121, V00.131, V00.141, V00.151, V00.181, V00.211, V00.221, V00.281, V00.311, V00.321, V00.381, V00.811, V00.821, V00.831, V00.841x, V00.891, V93.30x, V93.31x, V93.32x, V93.33x, V93.34x, V93.35x, V93.36x, V93.38x, V93.39x, W00.0xx, W00.1xx, W00.2xx, W00.9xx, W01.0xx, W01.10x, W01.110, W01.111, W01.118, W01.119, W01.190, W01.198, W03.xxx, W04.xxx, W05.0xx, W05.1xx, W05.2xx, W06.xxx, W07.xxx, W08.xxx, W09.0xx, W09.1xx, W09.2xx, W09.8xx, W10.0xx, W10.1xx, W10.2xx, W10.8xx, W10.9xx, W11.xxx, W12.xxx, W13.0xx, W13.1xx, W13.2xx, W13.3xx, W13.4xx, W13.8xx, W13.9xx, W14.xxx, W15.xxx, W16.011, W16.012, W16.021, W16.022, W16.031, W16.032, W16.111, W16.112, W16.121, W16.122, W16.131, W16.132, W16.211, W16.212, W16.221, W16.222, W16.311, W16.312, W16.321, W16.322, W16.331, W16.332, W16.41x, W16.42x, W16.511, W16.512, W16.521, W16.522, W16.531, W16.532, W16.611, W16.612, W16.621, W16.622, W16.711, W16.712, W16.721, W16.722, W16.811, W16.812, W16.821, W16.822, W16.831, W16.832, W16.91x, W16.92x, W17.0xx, W17.1xx, W17.2xx, W17.3xx, W17.4xx, W17.81x, W17.82x, W17.89x, W18.11x, W18.12x, W18.2xx, W18.30x, W18.31x, W18.39x, W18.40x, W18.41x, W18.42x, W18.43x, W18.49x, W19.xxx, X80.xxx, Y01.xxx, Y30.xxx |
| Fire/Burn | T54.1x1, T54.1x2, T54.1x3, T54.1x4, T54.2x1, T54.2x2, T54.2x3, T54.2x4, T54.3x1, T54.3x2, T54.3x3, T54.3x4, T54.91x, T54.92x, T54.93x, T54.94x, V91.00x, V91.01x, V91.02x, V91.03x, V91.04x, V91.05x, V91.06x, V91.07x, V91.08x, V91.09x, V91.10x, V93.00x, V93.01x, V93.02x, V93.03x, V93.04x, V93.09x, V93.10x, V93.11x, V93.12x, V93.13x, V93.14x, V93.19x, X00.0xx, X00.1xx, X00.2xx, X00.3xx, X00.4xx, X00.5xx, X00.8xx, X01.0xx, X01.1xx, X01.3xx, X01.4xx, X01.8xx, X02.0xx, X02.1xx, X02.2xx, X02.3xx, X02.4xx, X02.5xx, X02.8xx, X03.0xx, X03.1xx, X03.3xx, X03.4xx, X03.8xx, X04.xxx, X05.xxx, X06.0xx, X06.1xx, X06.2xx, X06.3xx, X08.00x, X08.01x, X08.09x, X08.10x, X08.11x, X08.19x, X08.20x, X08.21x, X08.29x, X08.8xx, X10.0xx, X10.1xx, X10.2xx, X11.0xx, X11.1xx, X11.8xx, X12.xxx, X13.0xx, X13.1xx, X14.0xx, X14.1xx, X15.0xx, X15.1xx, X15.2xx, X15.3xx, X15.8xx, X16.xxx, X17.xxx, X18.xxx, X19.xxx, X76.xxx, X77.0xx, X77.1xx, X77.2xx, X77.3xx, X77.8xx, X77.9xx, X97.xxx, X98.0xx, X98.1xx, X98.2xx, X98.3xx, X98.8xx, X98.9xx, Y26.xxx, Y27.0xx, Y27.1xx, Y27.2xx, Y27.3xx, Y27.8xx, Y27.9xx, Y36.300, Y36.301, Y36.330, Y36.331, Y36.390, Y36.391, Y37.300, Y37.301, Y37.330, Y37.331, Y37.390, Y37.391, Y38.3x1, Y38.3x2, Y38.3x3 |
| Firearm | W32.0xx, W32.1xx, W33.00x, W33.01x, W33.02x, W33.03x, W33.09x, W33.10x, W33.11x, W33.12x, W33.13x, W33.19x, W34.00x, W34.09x, W34.10x, W34.19x, X72.xxx, X73.0xx, X73.1xx, X73.2xx, X73.8xx, X73.9xx, X74.8xx, X74.9xx, X93.xxx, X94.0xx, X94.1xx, X94.2xx, X94.8xx, X94.9xx, X95.8xx, X95.9xx, Y22.xxx, Y23.0xx, Y23.1xx, Y23.2xx, Y23.3xx, Y23.8xx, Y23.9xx, Y24.8xx, Y24.9xx, Y35.001, Y35.002, Y35.003, Y35.009, Y35.011, Y35.012, Y35.013, Y35.019, Y35.021, Y35.022, Y35.023, Y35.029, Y35.031, Y35.032, Y35.033, Y35.039, Y35.091, Y35.092, Y35.093, Y35.099, Y36.420, Y36.421, Y36.430, Y36.431, Y36.92x, Y37.420, Y37.421, Y37.430, Y37.431, Y37.92x, Y38.4x1, Y38.4x2, Y38.4x3 |
| Machinery | V93.60x, V93.61x, V93.62x, V93.63x, V93.64x, V93.69x, W24.0xx, W24.1xx, W30.0xx, W30.1xx, W30.2xx, W30.3xx, W30.81x, W30.89x, W30.9xx, W31.0xx, W31.1xx, W31.2xx, W31.3xx, W31.81x, W31.82x, W31.83x, W31.89x, W31.9xx |
| Motor Vehicle-Nontraffic | V20.09x, V20.0xx, V20.19x, V20.1xx, V20.29x, V20.2xx, V20.39x, V21.09x, V21.0xx, V21.19x, V21.1xx, V21.29x, V21.2xx, V22.09x, V22.0xx, V22.19x, V22.1xx, V22.29x, V22.2xx, V23.09x, V23.0xx, V23.19x, V23.1xx, V23.29x, V23.2xx, V24.09x, V24.0xx, V24.19x, V24.1xx, V24.29x, V24.2xx, V25.09x, V25.0xx, V25.19x, V25.1xx, V25.29x, V25.2xx, V26.09x, V26.0xx, V26.19x, V26.1xx, V26.29x, V26.2xx, V27.09x, V27.0xx, V27.19x, V27.1xx, V27.29x, V27.2xx, V28.09x, V28.0xx, V28.19x, V28.1xx, V28.29x, V28.2xx, V28.39x, V28.49x, V28.59x, V28.99x, V29.008, V29.00x, V29.098, V29.09x, V29.108, V29.10x, V29.198, V29.19x, V29.208, V29.20x, V29.298, V29.29x, V29.39x, V29.3xx, V30.0xx, V30.1xx, V30.2xx, V30.3xx, V31.0xx, V31.1xx, V31.2xx, V31.3xx, V32.0xx, V32.1xx, V32.2xx, V32.3xx, V33.0xx, V33.1xx, V33.2xx, V33.3xx, V34.0xx, V34.1xx, V34.2xx, V34.3xx, V35.0xx, V35.1xx, V35.2xx, V35.3xx, V36.0xx, V36.1xx, V36.2xx, V36.3xx, V37.0xx, V37.1xx, V37.2xx, V37.3xx, V38.0xx, V38.1xx, V38.2xx, V38.3xx, V39.00x, V39.09x, V39.10x, V39.19x, V39.20x, V39.29x, V39.3xx, V40.0xx, V40.1xx, V40.2xx, V40.3xx, V41.0xx, V41.1xx, V41.2xx, V41.3xx, V42.0xx, V42.1xx, V42.2xx, V42.3xx, V43.01x, V43.02x, V43.03x, V43.04x, V43.11x, V43.12x, V43.13x, V43.14x, V43.21x, V43.22x, V43.23x, V43.24x, V43.31x, V43.32x, V43.33x, V43.34x, V44.0xx, V44.1xx, V44.2xx, V44.3xx, V45.0xx, V45.1xx, V45.2xx, V45.3xx, V46.0xx, V46.1xx, V46.2xx, V46.3xx, V47.01x, V47.02x, V47.0xx, V47.11x, V47.12x, V47.1xx, V47.2xx, V47.31x, V47.32x, V47.3xx, V48.0xx, V48.1xx, V48.2xx, V48.3xx, V49.00x, V49.09x, V49.10x, V49.19x, V49.20x, V49.29x, V49.3xx, V50.0xx, V50.1xx, V50.2xx, V50.3xx, V51.0xx, V51.1xx, V51.2xx, V51.3xx, V52.0xx, V52.1xx, V52.2xx, V52.3xx, V53.0xx, V53.1xx, V53.2xx, V53.3xx, V54.0xx, V54.1xx, V54.2xx, V54.3xx, V55.0xx, V55.1xx, V55.2xx, V55.3xx, V56.0xx, V56.1xx, V56.2xx, V56.3xx, V57.0xx, V57.1xx, V57.2xx, V57.3xx, V58.0xx, V58.1xx, V58.2xx, V58.3xx, V59.00x, V59.09x, V59.10x, V59.19x, V59.20x, V59.29x, V59.3xx, V60.0xx, V60.1xx, V60.2xx, V60.3xx, V61.0xx, V61.1xx, V61.2xx, V61.3xx, V62.0xx, V62.1xx, V62.2xx, V62.3xx, V63.0xx, V63.1xx, V63.2xx, V63.3xx, V64.0xx, V64.1xx, V64.2xx, V64.3xx, V65.0xx, V65.1xx, V65.2xx, V65.3xx, V66.0xx, V66.1xx, V66.2xx, V66.3xx, V67.0xx, V67.1xx, V67.2xx, V67.3xx, V68.0xx, V68.1xx, V68.2xx, V68.3xx, V69.00x, V69.09x, V69.10x, V69.19x, V69.20x, V69.29x, V69.3xx, V70.0xx, V70.1xx, V70.2xx, V70.3xx, V71.0xx, V71.1xx, V71.2xx, V71.3xx, V72.0xx, V72.1xx, V72.2xx, V72.3xx, V73.0xx, V73.1xx, V73.2xx, V73.3xx, V74.0xx, V74.1xx, V74.2xx, V74.3xx, V75.0xx, V75.1xx, V75.2xx, V75.3xx, V76.0xx, V76.1xx, V76.2xx, V76.3xx, V77.0xx, V77.1xx, V77.2xx, V77.3xx, V78.0xx, V78.1xx, V78.2xx, V78.3xx, V79.00x, V79.09x, V79.10x, V79.19x, V79.20x, V79.29x, V79.3xx, V81.0xx, V82.0xx, V84.5xx, V84.6xx, V84.7xx, V84.9xx, V85.5xx, V85.6xx, V85.7xx, V85.9xx, V86.51x, V86.54x, V86.55x, V86.56x, V86.61x, V86.64x, V86.65x, V86.66x, V86.71x, V86.74x, V86.75x, V86.76x, V86.91x, V86.94x, V86.95x, V86.96x, V88.0xx, V88.1xx, V88.2xx, V88.3xx, V88.4xx, V88.5xx, V88.6xx, V88.7xx, V88.8xx, V89.0xx |
| Motor Vehicle-Traffic | V02.10x, V02.11x, V02.12x, V02.13x, V02.19x, V02.90x, V02.91x, V02.92x, V02.93x, V02.99x, V03.10x, V03.11x, V03.12x, V03.13x, V03.19x, V03.90x, V03.91x, V03.92x, V03.93x, V03.99x, V04.10x, V04.11x, V04.12x, V04.13x, V04.19x, V04.90x, V04.91x, V04.92x, V04.93x, V04.99x, V05.10x, V05.11x, V05.12x, V05.13x, V05.19x, V05.90x, V05.91x, V05.92x, V05.93x, V05.99x, V09.20x, V09.21x, V09.29x, V09.3xx, V12.3xx, V12.4xx, V12.5xx, V12.9xx, V13.3xx, V13.4xx, V13.5xx, V13.9xx, V14.3xx, V14.4xx, V14.5xx, V14.9xx, V19.40x, V19.49x, V19.50x, V19.59x, V19.60x, V19.69x, V19.9xx, V20.3xx, V20.49x, V20.4xx, V20.59x, V20.5xx, V20.99x, V20.9xx, V21.39x, V21.3xx, V21.49x, V21.4xx, V21.59x, V21.5xx, V21.99x, V21.9xx, V22.31x, V22.39x, V22.3xx, V22.41x, V22.49x, V22.4xx, V22.51x, V22.59x, V22.5xx, V22.91x, V22.99x, V22.9xx, V23.31x, V23.39x, V23.3xx, V23.41x, V23.49x, V23.4xx, V23.51x, V23.59x, V23.5xx, V23.91x, V23.99x, V23.9xx, V24.31x, V24.39x, V24.3xx, V24.41x, V24.49x, V24.4xx, V24.51x, V24.59x, V24.5xx, V24.91x, V24.99x, V24.9xx, V25.31x, V25.39x, V25.3xx, V25.41x, V25.49x, V25.4xx, V25.51x, V25.59x, V25.5xx, V25.91x, V25.99x, V25.9xx, V26.39x, V26.3xx, V26.49x, V26.4xx, V26.59x, V26.5xx, V26.99x, V26.9xx, V27.39x, V27.3xx, V27.49x, V27.4xx, V27.59x, V27.5xx, V27.99x, V27.9xx, V28.3xx, V28.4xx, V28.5xx, V28.9xx, V29.401, V29.408, V29.40x, V29.491, V29.498, V29.49x, V29.501, V29.508, V29.50x, V29.591, V29.598, V29.59x, V29.601, V29.608, V29.60x, V29.691, V29.698, V29.69x, V29.818, V29.81x, V29.888, V29.88x, V29.99x, V29.9xx, V30.4xx, V30.5xx, V30.6xx, V30.7xx, V30.9xx, V31.4xx, V31.5xx, V31.6xx, V31.7xx, V31.9xx, V32.4xx, V32.5xx, V32.6xx, V32.7xx, V32.9xx, V33.4xx, V33.5xx, V33.6xx, V33.7xx, V33.9xx, V34.4xx, V34.5xx, V34.6xx, V34.7xx, V34.9xx, V35.4xx, V35.5xx, V35.6xx, V35.7xx, V35.9xx, V36.4xx, V36.5xx, V36.6xx, V36.7xx, V36.9xx, V37.4xx, V37.5xx, V37.6xx, V37.7xx, V37.9xx, V38.4xx, V38.5xx, V38.6xx, V38.7xx, V38.9xx, V39.40x, V39.49x, V39.50x, V39.59x, V39.60x, V39.69x, V39.81x, V39.89x, V39.9xx, V40.4xx, V40.5xx, V40.6xx, V40.7xx, V40.9xx, V41.4xx, V41.5xx, V41.6xx, V41.7xx, V41.9xx, V42.4xx, V42.5xx, V42.6xx, V42.7xx, V42.9xx, V43.41x, V43.42x, V43.43x, V43.44x, V43.51x, V43.52x, V43.53x, V43.54x, V43.61x, V43.62x, V43.63x, V43.64x, V43.71x, V43.72x, V43.73x, V43.74x, V43.91x, V43.92x, V43.93x, V43.94x, V44.4xx, V44.5xx, V44.6xx, V44.7xx, V44.9xx, V45.4xx, V45.5xx, V45.6xx, V45.7xx, V45.9xx, V46.4xx, V46.5xx, V46.6xx, V46.7xx, V46.9xx, V47.4xx, V47.51x, V47.52x, V47.5xx, V47.61x, V47.62x, V47.6xx, V47.7xx, V47.91x, V47.92x, V47.9xx, V48.4xx, V48.5xx, V48.6xx, V48.7xx, V48.9xx, V49.40x, V49.49x, V49.50x, V49.59x, V49.60x, V49.69x, V49.81x, V49.88x, V49.9xx, V50.4xx, V50.5xx, V50.6xx, V50.7xx, V50.9xx, V51.4xx, V51.5xx, V51.6xx, V51.7xx, V51.9xx, V52.4xx, V52.5xx, V52.6xx, V52.7xx, V52.9xx, V53.4xx, V53.5xx, V53.6xx, V53.7xx, V53.9xx, V54.4xx, V54.5xx, V54.6xx, V54.7xx, V54.9xx, V55.4xx, V55.5xx, V55.6xx, V55.7xx, V55.9xx, V56.4xx, V56.5xx, V56.6xx, V56.7xx, V56.9xx, V57.4xx, V57.5xx, V57.6xx, V57.7xx, V57.9xx, V58.4xx, V58.5xx, V58.6xx, V58.7xx, V58.9xx, V59.40x, V59.49x, V59.50x, V59.59x, V59.60x, V59.69x, V59.81x, V59.88x, V59.9xx, V60.4xx, V60.5xx, V60.6xx, V60.7xx, V60.9xx, V61.4xx, V61.5xx, V61.6xx, V61.7xx, V61.9xx, V62.4xx, V62.5xx, V62.6xx, V62.7xx, V62.9xx, V63.4xx, V63.5xx, V63.6xx, V63.7xx, V63.9xx, V64.4xx, V64.5xx, V64.6xx, V64.7xx, V64.9xx, V65.4xx, V65.5xx, V65.6xx, V65.7xx, V65.9xx, V66.4xx, V66.5xx, V66.6xx, V66.7xx, V66.9xx, V67.4xx, V67.5xx, V67.6xx, V67.7xx, V67.9xx, V68.4xx, V68.5xx, V68.6xx, V68.7xx, V68.9xx, V69.40x, V69.49x, V69.50x, V69.59x, V69.60x, V69.69x, V69.81x, V69.88x, V69.9xx, V70.4xx, V70.5xx, V70.6xx, V70.7xx, V70.9xx, V71.4xx, V71.5xx, V71.6xx, V71.7xx, V71.9xx, V72.4xx, V72.5xx, V72.6xx, V72.7xx, V72.9xx, V73.4xx, V73.5xx, V73.6xx, V73.7xx, V73.9xx, V74.4xx, V74.5xx, V74.6xx, V74.7xx, V74.9xx, V75.4xx, V75.5xx, V75.6xx, V75.7xx, V75.9xx, V76.4xx, V76.5xx, V76.6xx, V76.7xx, V76.9xx, V77.4xx, V77.5xx, V77.6xx, V77.7xx, V77.9xx, V78.4xx, V78.5xx, V78.6xx, V78.7xx, V78.9xx, V79.40x, V79.49x, V79.50x, V79.59x, V79.60x, V79.69x, V79.81x, V79.88x, V79.9xx, V80.31x, V80.32x, V80.41x, V80.42x, V80.51x, V80.52x, V81.1xx, V82.1xx, V83.0xx, V83.1xx, V83.2xx, V83.3xx, V84.0xx, V84.1xx, V84.2xx, V84.3xx, V85.0xx, V85.1xx, V85.2xx, V85.3xx, V86.01x, V86.02x, V86.03x, V86.04x, V86.05x, V86.06x, V86.09x, V86.11x, V86.12x, V86.13x, V86.14x, V86.15x, V86.16x, V86.19x, V86.21x, V86.22x, V86.23x, V86.24x, V86.25x, V86.26x, V86.29x, V86.31x, V86.32x, V86.33x, V86.34x, V86.35x, V86.36x, V86.39x, V87.0xx, V87.1xx, V87.2xx, V87.3xx, V87.4xx, V87.5xx, V87.6xx, V87.7xx, V87.8xx, V89.2xx, X81.0xx, X82.0xx, X82.1xx, X82.2xx, X82.8xx, Y02.0xx, Y03.0xx, Y03.8xx, Y32.xxx |
| Natural/Environmental | T63.001, T63.002, T63.003, T63.004, T63.011, T63.012, T63.013, T63.014, T63.021, T63.022, T63.023, T63.024, T63.031, T63.032, T63.033, T63.034, T63.041, T63.042, T63.043, T63.044, T63.061, T63.062, T63.063, T63.064, T63.071, T63.072, T63.073, T63.074, T63.081, T63.082, T63.083, T63.084, T63.091, T63.092, T63.093, T63.094, T63.111, T63.112, T63.113, T63.114, T63.121, T63.122, T63.123, T63.124, T63.191, T63.192, T63.193, T63.194, T63.2x1, T63.2x2, T63.2x3, T63.2x4, T63.301, T63.302, T63.303, T63.304, T63.311, T63.312, T63.313, T63.314, T63.321, T63.322, T63.323, T63.324, T63.331, T63.332, T63.333, T63.334, T63.391, T63.392, T63.393, T63.394, T63.411, T63.412, T63.413, T63.414, T63.421, T63.422, T63.423, T63.424, T63.431, T63.432, T63.433, T63.434, T63.441, T63.442, T63.443, T63.444, T63.451, T63.452, T63.453, T63.454, T63.461, T63.462, T63.463, T63.464, T63.481, T63.482, T63.483, T63.484, T63.511, T63.512, T63.513, T63.514, T63.591, T63.592, T63.593, T63.594, T63.611, T63.612, T63.613, T63.614, T63.621, T63.622, T63.623, T63.624, T63.631, T63.632, T63.633, T63.634, T63.691, T63.692, T63.693, T63.694, T63.711, T63.712, T63.713, T63.714, T63.791, T63.792, T63.793, T63.794, T63.811, T63.812, T63.813, T63.814, T63.821, T63.822, T63.823, T63.824, T63.831, T63.832, T63.833, T63.834, T63.891, T63.892, T63.893, T63.894, T63.91x, T63.92x, T63.93x, T63.94x, T65.821, T65.822, T65.823, T65.824, T73.0xx, T73.1xx, T73.2xx, T73.8xx, T73.9xx, T75.00x, T75.01x, T75.09x, T75.0xx, T75.20x, T75.21x, T75.22x, T75.23x, T75.29x, T75.2xx, T75.3xx, V93.20x, V93.21x, V93.22x, V93.23x, V93.24x, V93.29x, W42.0xx, W42.9xx, W53.01x, W53.09x, W53.11x, W53.19x, W53.21x, W53.29x, W53.81x, W53.89x, W54.0xx, W54.1xx, W54.8xx, W55.01x, W55.03x, W55.09x, W55.11x, W55.12x, W55.19x, W55.21x, W55.22x, W55.29x, W55.31x, W55.32x, W55.39x, W55.41x, W55.42x, W55.49x, W55.51x, W55.52x, W55.59x, W55.81x, W55.82x, W55.89x, W56.01x, W56.02x, W56.09x, W56.11x, W56.12x, W56.19x, W56.21x, W56.22x, W56.29x, W56.31x, W56.32x, W56.39x, W56.41x, W56.42x, W56.49x, W56.51x, W56.52x, W56.59x, W56.81x, W56.82x, W56.89x, W57.xxx, W58.01x, W58.02x, W58.03x, W58.09x, W58.11x, W58.12x, W58.13x, W58.19x, W59.01x, W59.02x, W59.09x, W59.11x, W59.12x, W59.13x, W59.19x, W59.21x, W59.22x, W59.29x, W59.81x, W59.82x, W59.83x, W59.89x, W60.xxx, W61.01x, W61.02x, W61.09x, W61.11x, W61.12x, W61.19x, W61.21x, W61.22x, W61.29x, W61.32x, W61.33x, W61.39x, W61.42x, W61.43x, W61.49x, W61.51x, W61.52x, W61.59x, W61.61x, W61.62x, W61.69x, W61.91x, W61.92x, W61.99x, W62.0xx, W62.1xx, W62.9xx, W64.xxx, W92.xxx, W93.01x, W93.02x, W93.11x, W93.12x, W93.2xx, W93.8xx, W94.0xx, W94.11x, W94.12x, W94.21x, W94.22x, W94.23x, W94.29x, W94.31x, W94.32x, W94.39x, W99.xxx, X30.xxx, X31.xxx, X32.xxx, X34.xxx, X35.xxx, X36.0xx, X36.1xx, X37.0xx, X37.1xx, X37.2xx, X37.3xx, X37.41x, X37.42x, X37.43x, X37.8xx, X37.9xx, X38.xxx, X39.01x, X39.08x, X39.8xx, X52.xxx, X83.2xx |
| Other Land Transport | V80.010, V80.018, V80.02x, V80.11x, V80.12x, V80.21x, V80.22x, V80.61x, V80.62x, V80.710, V80.711, V80.720, V80.721, V80.730, V80.731, V80.790, V80.791, V80.81x, V80.82x, V80.910, V80.918, V80.919, V80.920, V80.928, V80.929, V81.2xx, V81.3xx, V81.4xx, V81.5xx, V81.6xx, V81.7xx, V81.81x, V81.82x, V81.83x, V81.89x, V81.9xx, V82.2xx, V82.3xx, V82.4xx, V82.5xx, V82.6xx, V82.7xx, V82.8xx, V82.9xx, V83.4xx, V83.5xx, V83.6xx, V83.7xx, V83.9xx, V84.4xx, V85.4xx, V86.41x, V86.42x, V86.43x, V86.44x, V86.45x, V86.46x, V86.49x, V86.52x, V86.53x, V86.59x, V86.62x, V86.63x, V86.69x, V86.72x, V86.73x, V86.79x, V86.92x, V86.93x, V86.99x, V87.9xx, V88.9xx, V89.1xx, V89.3xx, V89.9xx, X81.1xx, Y02.1xx |
| Other Specified | T15.00x, T15.01x, T15.02x, T15.10x, T15.11x, T15.12x, T15.80x, T15.81x, T15.82x, T15.90x, T15.91x, T15.92x, T16.1xx, T16.2xx, T16.9xx, T17.0xx, T17.1xx, T17.208, T17.218, T17.228, T17.298, T17.308, T17.318, T17.328, T17.398, T17.408, T17.418, T17.428, T17.498, T17.508, T17.518, T17.528, T17.598, T17.808, T17.818, T17.828, T17.898, T17.908, T17.918, T17.928, T17.998, T18.0xx, T18.100, T18.108, T18.110, T18.118, T18.120, T18.128, T18.190, T18.198, T18.2xx, T18.3xx, T18.4xx, T18.5xx, T18.8xx, T18.9xx, T19.0xx, T19.1xx, T19.2xx, T19.3xx, T19.4xx, T19.8xx, T19.9xx, T74.01x , T74.02x , T74.11x , T74.12x , T74.21x , T74.22x, T74.31x , T74.32x , T74.4xx , T74.51, T74.52, T74.61, T74.62, T74.91x , T74.92x , T75.4xx, T76.01x , T76.02x , T76.11x , T76.12x , T76.21x , T76.22x , T76.31x , T76.32x , T76.51, T76.52, T76.61, T76.62, T76.91x , T76.92x, V00.118, V00.128, V00.138, V00.148, V00.158, V00.188, V00.218, V00.228, V00.288, V00.318, V00.328, V00.388, V00.818, V00.828, V00.838, V00.848X, V00.898, W23.0xx, W23.1xx, W23.2xx, W34.010, W34.011, W34.018, W34.110, W34.111, W34.118, W35.xxx, W36.1xx, W36.2xx, W36.3xx, W36.8xx, W36.9xx, W37.0xx, W37.8xx, W38.xxx, W39.xxx, W40.0xx, W40.1xx, W40.8xx, W40.9xx, W44.8xx, W44.9xx, W44.A0xx, W44.A1xx, W44.A9xx, W44.B0xx, W44.B1xx, W44.B2xx, W44.B3xx, W44.B4xx, W44.B5xx, W44.B9xx, W44.C0xx, W44.C1xx, W44.C2xx, W44.D0xx, W44.D1xx, W44.D2xx, W44.D3xx, W44.D4xx, W44.D9xx, W44.E0xx, W44.E1xx, W44.E2xx, W44.E3xx, W44.E4xx, W44.E9xx, W44.F0xx, W44.F1xx, W44.F2xx, W44.F3xx, W44.F4xx, W44.F9xx, W44.G0xx, W44.G1xx, W44.G2xx, W44.G3xx, W44.G9xx, W44.H0xx, W44.H1xx, W44.H2xx, W49.01x, W49.02x, W49.03x, W49.04x, W49.09x, W49.9xx, W85.xxx, W86.0xx, W86.1xx, W86.8xx, W88.0xx, W88.1xx, W88.8xx, W89.0xx, W89.1xx, W89.8xx, W89.9xx, W90.0xx, W90.1xx, W90.2xx, W90.8xx, X74.01x, X74.02x, X74.09x, X75.xxx, X81.8xx, X83.1xx, X83.8xx, X95.01x, X95.02x, X95.09x, X96.0xx, X96.1xx, X96.2xx, X96.3xx, X96.4xx, X96.8xx, X96.9xx, Y02.8xx, Y07.01, Y07.02, Y07.03, Y07.04, Y07.11, Y07.12, Y07.13, Y07.14, Y07.410, Y07.411, Y07.420, Y07.421, Y07.430, Y07.432, Y07.433, Y07.434, Y07.435, Y07.436, Y07.490, Y07.491, Y07.499, Y07.50, Y07.510, Y07.511, Y07.512, Y07.513, Y07.519, Y07.521, Y07.528, Y07.529, Y07.53, Y07.59, Y07.6, Y07.9, Y08.89x, Y24.0xx, Y25.xxx, Y31.xxx, Y33.xxx, Y35.041, Y35.042, Y35.043, Y35.049, Y35.101, Y35.102, Y35.103, Y35.109, Y35.111, Y35.112, Y35.113, Y35.119, Y35.121, Y35.122, Y35.123, Y35.129, Y35.191, Y35.192, Y35.193, Y35.199, Y35.831x, Y35.832x, Y35.833x, Y35.839x, Y35.891, Y35.892, Y35.893, Y35.899, Y36.000, Y36.001, Y36.010, Y36.011, Y36.020, Y36.021, Y36.030, Y36.031, Y36.040, Y36.041, Y36.050, Y36.051, Y36.090, Y36.091, Y36.200, Y36.201, Y36.210, Y36.211, Y36.220, Y36.221, Y36.230, Y36.231, Y36.240, Y36.241, Y36.250, Y36.251, Y36.260, Y36.261, Y36.270, Y36.271, Y36.290, Y36.291, Y36.310, Y36.311, Y36.320, Y36.321, Y36.410, Y36.411, Y36.450, Y36.451, Y36.490, Y36.491, Y36.500, Y36.501, Y36.510, Y36.511, Y36.520, Y36.521, Y36.530, Y36.531, Y36.540, Y36.541, Y36.590, Y36.591, Y36.6x0, Y36.6x1, Y36.810, Y36.811, Y36.820, Y36.821, Y36.880, Y36.881, Y36.91x, Y37.000, Y37.001, Y37.010, Y37.011, Y37.020, Y37.021, Y37.030, Y37.031, Y37.040, Y37.041, Y37.050, Y37.051, Y37.090, Y37.091, Y37.200, Y37.201, Y37.210, Y37.211, Y37.220, Y37.221, Y37.230, Y37.231, Y37.240, Y37.241, Y37.250, Y37.251, Y37.260, Y37.261, Y37.270, Y37.271, Y37.290, Y37.291, Y37.310, Y37.311, Y37.320, Y37.321, Y37.410, Y37.411, Y37.450, Y37.451, Y37.490, Y37.491, Y37.500, Y37.501, Y37.510, Y37.511, Y37.520, Y37.521, Y37.530, Y37.531, Y37.540, Y37.541, Y37.590, Y37.591, Y37.6x0, Y37.6x1, Y37.91x, Y38.0x1, Y38.0x2, Y38.0x3, Y38.2x1, Y38.2x2, Y38.2x3, Y38.5x1, Y38.5x2, Y38.5x3, Y38.6x1, Y38.6x2, Y38.6x3, Y38.811, Y38.812, Y38.891, Y38.892, Y38.893, Y38.9x1, Y38.9x2 |
| Other Transport | V90.00x, V90.01x, V90.02x, V90.03x, V90.04x, V90.05x, V90.06x, V90.08x, V90.09x, V90.10x, V90.11x, V90.12x, V90.13x, V90.14x, V90.15x, V90.16x, V90.18x, V90.19x, V90.20x, V90.21x, V90.22x, V90.23x, V90.24x, V90.25x, V90.26x, V90.27x, V90.28x, V90.29x, V90.30x, V90.31x, V90.32x, V90.33x, V90.34x, V90.35x, V90.36x, V90.37x, V90.38x, V90.39x, V90.80x, V90.81x, V90.82x, V90.83x, V90.84x, V90.85x, V90.86x, V90.87x, V90.88x, V90.89x, V91.11x, V91.12x, V91.13x, V91.14x, V91.15x, V91.16x, V91.18x, V91.19x, V91.20x, V91.21x, V91.22x, V91.23x, V91.24x, V91.25x, V91.26x, V91.29x, V91.30x, V91.31x, V91.32x, V91.33x, V91.34x, V91.35x, V91.36x, V91.37x, V91.38x, V91.39x, V91.80x, V91.81x, V91.82x, V91.83x, V91.84x, V91.85x, V91.86x, V91.87x, V91.88x, V91.89x, V92.00x, V92.01x, V92.02x, V92.03x, V92.04x, V92.05x, V92.06x, V92.07x, V92.08x, V92.09x, V92.10x, V92.11x, V92.12x, V92.13x, V92.14x, V92.15x, V92.16x, V92.19x, V92.20x, V92.21x, V92.22x, V92.23x, V92.24x, V92.25x, V92.26x, V92.27x, V92.28x, V92.29x, V93.50x, V93.51x, V93.52x, V93.53x, V93.54x, V93.59x, V93.80x, V93.81x, V93.82x, V93.83x, V93.84x, V93.85x, V93.86x, V93.87x, V93.88x, V93.89x, V94.0xx, V94.11x, V94.12x, V94.21x, V94.22x, V94.31x, V94.32x, V94.4xx, V94.810, V94.811, V94.818, V94.89x, V94.9xx, V95.00x, V95.01x, V95.02x, V95.03x, V95.04x, V95.05x, V95.09x, V95.10x, V95.11x, V95.12x, V95.13x, V95.14x, V95.15x, V95.19x, V95.20x, V95.21x, V95.22x, V95.23x, V95.24x, V95.25x, V95.29x, V95.30x, V95.31x, V95.32x, V95.33x, V95.34x, V95.35x, V95.39x, V95.40x, V95.41x, V95.42x, V95.43x, V95.44x, V95.45x, V95.49x, V95.8xx, V95.9xx, V96.00x, V96.01x, V96.02x, V96.03x, V96.04x, V96.05x, V96.09x, V96.10x, V96.11x, V96.12x, V96.13x, V96.14x, V96.15x, V96.19x, V96.20x, V96.21x, V96.22x, V96.23x, V96.24x, V96.25x, V96.29x, V96.8xx, V96.9xx, V97.0xx, V97.1xx, V97.21x, V97.22x, V97.29x, V97.31x, V97.32x, V97.33x, V97.39x, V97.810, V97.811, V97.818, V97.89x, V98.0xx, V98.1xx, V98.2xx, V98.3xx, V98.8xx, V99.xxx, X83.0xx, Y08.81x, Y36.100, Y36.101, Y36.110, Y36.111, Y36.120, Y36.121, Y36.130, Y36.131, Y36.140, Y36.141, Y36.190, Y36.191, Y37.100, Y37.101, Y37.110, Y37.111, Y37.120, Y37.121, Y37.130, Y37.131, Y37.140, Y37.141, Y37.190, Y37.191, Y38.1x1, Y38.1x2, Y38.1x3 |
| Overexertion | T73.3xx, X50.0xx, X50.1xx, X50.3xx, X50.9xx |
| Pedal cyclist, other | V10.0xx, V10.1xx, V10.2xx, V10.3xx, V10.4xx, V10.5xx, V10.9xx, V11.0xx, V11.1xx, V11.2xx, V11.3xx, V11.4xx, V11.5xx, V11.9xx, V12.0xx, V12.1xx, V12.2xx, V13.0xx, V13.1xx, V13.2xx, V14.0xx, V14.1xx, V14.2xx, V15.0xx, V15.1xx, V15.2xx, V15.3xx, V15.4xx, V15.5xx, V15.9xx, V16.0xx, V16.1xx, V16.2xx, V16.3xx, V16.4xx, V16.5xx, V16.9xx, V17.0xx, V17.1xx, V17.2xx, V17.3xx, V17.4xx, V17.5xx, V17.9xx, V18.0xx, V18.1xx, V18.2xx, V18.3xx, V18.4xx, V18.5xx, V18.9xx, V19.00x, V19.09x, V19.10x, V19.19x, V19.20x, V19.29x, V19.3xx, V19.81x, V19.88x, V20.01x, V20.11x, V20.21x, V20.31x, V20.41x, V20.51x, V20.91x, V21.01x, V21.11x, V21.21x, V21.31x, V21.41x, V21.51x, V21.91x, V22.01x, V22.11x, V22.21x, V23.01x, V23.11x, V23.21x, V24.01x, V24.11x, V24.21x, V25.01x, V25.11x, V25.21x, V26.01x, V26.11x, V26.21x, V26.31x, V26.41x, V26.51x, V26.91x, V27.01x, V27.11x, V27.21x, V27.31x, V27.41x, V27.51x, V27.91x, V28.01x, V28.11x, V28.21x, V28.31x, V28.41x, V28.51x, V28.91x, V29.001, V29.091, V29.101, V29.191, V29.201, V29.291, V29.31x, V29.811, V29.881, V29.91x |
| Pedestrian, other | V01.00x, V01.01x, V01.02x, V01.03x, V01.09x, V01.10x, V01.11x, V01.12x, V01.13x, V01.19x, V01.90x, V01.91x, V01.92x, V01.93x, V01.99x, V02.00x, V02.01x, V02.02x, V02.03x, V02.09x, V03.00x, V03.01x, V03.02x, V03.03x, V03.09x, V04.00x, V04.01x, V04.02x, V04.03x, V04.09x, V05.00x, V05.01x, V05.02x, V05.03x, V05.09x, V06.00x, V06.01x, V06.02x, V06.03x, V06.09x, V06.10x, V06.11x, V06.12x, V06.13x, V06.19x, V06.90x, V06.91x, V06.92x, V06.93x, V06.99x, V09.00x, V09.01x, V09.09x, V09.1xx, V09.9xx |
| Poisoning | T36.0x1, T36.0x2, T36.0x3, T36.0x4, T36.1x1, T36.1x2, T36.1x3, T36.1x4, T36.2x1, T36.2x2, T36.2x3, T36.2x4, T36.3x1, T36.3x2, T36.3x3, T36.3x4, T36.4x1, T36.4x2, T36.4x3, T36.4x4, T36.5x1, T36.5x2, T36.5x3, T36.5x4, T36.6x1, T36.6x2, T36.6x3, T36.6x4, T36.7x1, T36.7x2, T36.7x3, T36.7x4, T36.8x1, T36.8x2, T36.8x3, T36.8x4, T36.91x, T36.92x, T36.93x, T36.94x, T37.0x1, T37.0x2, T37.0x3, T37.0x4, T37.1x1, T37.1x2, T37.1x3, T37.1x4, T37.2x1, T37.2x2, T37.2x3, T37.2x4, T37.3x1, T37.3x2, T37.3x3, T37.3x4, T37.4x1, T37.4x2, T37.4x3, T37.4x4, T37.5x1, T37.5x2, T37.5x3, T37.5x4, T37.8x1, T37.8x2, T37.8x3, T37.8x4, T37.91x, T37.92x, T37.93x, T37.94x, T38.0x1, T38.0x2, T38.0x3, T38.0x4, T38.1x1, T38.1x2, T38.1x3, T38.1x4, T38.2x1, T38.2x2, T38.2x3, T38.2x4, T38.3x1, T38.3x2, T38.3x3, T38.3x4, T38.4x1, T38.4x2, T38.4x3, T38.4x4, T38.5x1, T38.5x2, T38.5x3, T38.5x4, T38.6x1, T38.6x2, T38.6x3, T38.6x4, T38.7x1, T38.7x2, T38.7x3, T38.7x4, T38.801, T38.802, T38.803, T38.804, T38.811, T38.812, T38.813, T38.814, T38.891, T38.892, T38.893, T38.894, T38.901, T38.902, T38.903, T38.904, T38.991, T38.992, T38.993, T38.994, T39.011, T39.012, T39.013, T39.014, T39.091, T39.092, T39.093, T39.094, T39.1x1, T39.1x2, T39.1x3, T39.1x4, T39.2x1, T39.2x2, T39.2x3, T39.2x4, T39.311, T39.312, T39.313, T39.314, T39.391, T39.392, T39.393, T39.394, T39.4x1, T39.4x2, T39.4x3, T39.4x4, T39.8x1, T39.8x2, T39.8x3, T39.8x4, T39.91x, T39.92x, T39.93x, T39.94x, T40.0x1, T40.0x2, T40.0x3, T40.0x4, T40.1x1, T40.1x2, T40.1x3, T40.1x4, T40.2x1, T40.2x2, T40.2x3, T40.2x4, T40.3x1, T40.3x2, T40.3x3, T40.3x4, T40.411, T40.412, T40.413, T40.414, T40.421, T40.422, T40.423, T40.424, T40.491, T40.492, T40.493, T40.494, T40.4x1, T40.4x2, T40.4x3, T40.4x4, T40.5x1, T40.5x2, T40.5x3, T40.5x4, T40.601, T40.602, T40.603, T40.604, T40.691, T40.692, T40.693, T40.694, T40.711, T40.712, T40.713, T40.714, T40.721, T40.722, T40.723, T40.724, T40.7x1, T40.7x2, T40.7x3, T40.7x4, T40.8x1, T40.8x2, T40.8x3, T40.8x4, T40.901, T40.902, T40.903, T40.904, T40.991, T40.992, T40.993, T40.994, T41.0x1, T41.0x2, T41.0x3, T41.0x4, T41.1x1, T41.1x2, T41.1x3, T41.1x4, T41.201, T41.202, T41.203, T41.204, T41.291, T41.292, T41.293, T41.294, T41.3x1, T41.3x2, T41.3x3, T41.3x4, T41.41x, T41.42x, T41.43x, T41.44x, T41.5x1, T41.5x2, T41.5x3, T41.5x4, T42.0x1, T42.0x2, T42.0x3, T42.0x4, T42.1x1, T42.1x2, T42.1x3, T42.1x4, T42.2x1, T42.2x2, T42.2x3, T42.2x4, T42.3x1, T42.3x2, T42.3x3, T42.3x4, T42.4x1, T42.4x2, T42.4x3, T42.4x4, T42.5x1, T42.5x2, T42.5x3, T42.5x4, T42.6x1, T42.6x2, T42.6x3, T42.6x4, T42.71x, T42.72x, T42.73x, T42.74x, T42.8x1, T42.8x2, T42.8x3, T42.8x4, T43.011, T43.012, T43.013, T43.014, T43.021, T43.022, T43.023, T43.024, T43.1x1, T43.1x2, T43.1x3, T43.1x4, T43.201, T43.202, T43.203, T43.204, T43.211, T43.212, T43.213, T43.214, T43.221, T43.222, T43.223, T43.224, T43.291, T43.292, T43.293, T43.294, T43.3x1, T43.3x2, T43.3x3, T43.3x4, T43.4x1, T43.4x2, T43.4x3, T43.4x4, T43.501, T43.502, T43.503, T43.504, T43.591, T43.592, T43.593, T43.594, T43.601, T43.602, T43.603, T43.604, T43.611, T43.612, T43.613, T43.614, T43.621, T43.622, T43.623, T43.624, T43.631, T43.632, T43.633, T43.634, T43.641, T43.642, T43.643, T43.644, T43.691, T43.692, T43.693, T43.694, T43.8x1, T43.8x2, T43.8x3, T43.8x4, T43.91x, T43.92x, T43.93x, T43.94x, T44.0x1, T44.0x2, T44.0x3, T44.0x4, T44.1x1, T44.1x2, T44.1x3, T44.1x4, T44.2x1, T44.2x2, T44.2x3, T44.2x4, T44.3x1, T44.3x2, T44.3x3, T44.3x4, T44.4x1, T44.4x2, T44.4x3, T44.4x4, T44.5x1, T44.5x2, T44.5x3, T44.5x4, T44.6x1, T44.6x2, T44.6x3, T44.6x4, T44.7x1, T44.7x2, T44.7x3, T44.7x4, T44.8x1, T44.8x2, T44.8x3, T44.8x4, T44.901, T44.902, T44.903, T44.904, T44.991, T44.992, T44.993, T44.994, T45.0x1, T45.0x2, T45.0x3, T45.0x4, T45.1x1, T45.1x2, T45.1x3, T45.1x4, T45.2x1, T45.2x2, T45.2x3, T45.2x4, T45.3x1, T45.3x2, T45.3x3, T45.3x4, T45.4x1, T45.4x2, T45.4x3, T45.4x4, T45.511, T45.512, T45.513, T45.514, T45.521, T45.522, T45.523, T45.524, T45.601, T45.602, T45.603, T45.604, T45.611, T45.612, T45.613, T45.614, T45.621, T45.622, T45.623, T45.624, T45.691, T45.692, T45.693, T45.694, T45.7x1, T45.7x2, T45.7x3, T45.7x4, T45.8x1, T45.8x2, T45.8x3, T45.8x4, T45.91x, T45.92x, T45.93x, T45.94x, T46.0x1, T46.0x2, T46.0x3, T46.0x4, T46.1x1, T46.1x2, T46.1x3, T46.1x4, T46.2x1, T46.2x2, T46.2x3, T46.2x4, T46.3x1, T46.3x2, T46.3x3, T46.3x4, T46.4x1, T46.4x2, T46.4x3, T46.4x4, T46.5x1, T46.5x2, T46.5x3, T46.5x4, T46.6x1, T46.6x2, T46.6x3, T46.6x4, T46.7x1, T46.7x2, T46.7x3, T46.7x4, T46.8x1, T46.8x2, T46.8x3, T46.8x4, T46.901, T46.902, T46.903, T46.904, T46.991, T46.992, T46.993, T46.994, T47.0x1, T47.0x2, T47.0x3, T47.0x4, T47.1x1, T47.1x2, T47.1x3, T47.1x4, T47.2x1, T47.2x2, T47.2x3, T47.2x4, T47.3x1, T47.3x2, T47.3x3, T47.3x4, T47.4x1, T47.4x2, T47.4x3, T47.4x4, T47.5x1, T47.5x2, T47.5x3, T47.5x4, T47.6x1, T47.6x2, T47.6x3, T47.6x4, T47.7x1, T47.7x2, T47.7x3, T47.7x4, T47.8x1, T47.8x2, T47.8x3, T47.8x4, T47.91x, T47.92x, T47.93x, T47.94x, T48.0x1, T48.0x2, T48.0x3, T48.0x4, T48.1x1, T48.1x2, T48.1x3, T48.1x4, T48.201, T48.202, T48.203, T48.204, T48.291, T48.292, T48.293, T48.294, T48.3x1, T48.3x2, T48.3x3, T48.3x4, T48.4x1, T48.4x2, T48.4x3, T48.4x4, T48.5x1, T48.5x2, T48.5x3, T48.5x4, T48.6x1, T48.6x2, T48.6x3, T48.6x4, T48.901, T48.902, T48.903, T48.904, T48.991, T48.992, T48.993, T48.994, T49.0x1, T49.0x2, T49.0x3, T49.0x4, T49.1x1, T49.1x2, T49.1x3, T49.1x4, T49.2x1, T49.2x2, T49.2x3, T49.2x4, T49.3x1, T49.3x2, T49.3x3, T49.3x4, T49.4x1, T49.4x2, T49.4x3, T49.4x4, T49.5x1, T49.5x2, T49.5x3, T49.5x4, T49.6x1, T49.6x2, T49.6x3, T49.6x4, T49.7x1, T49.7x2, T49.7x3, T49.7x4, T49.8x1, T49.8x2, T49.8x3, T49.8x4, T49.91x, T49.92x, T49.93x, T49.94x, T50.0x1, T50.0x2, T50.0x3, T50.0x4, T50.1x1, T50.1x2, T50.1x3, T50.1x4, T50.2x1, T50.2x2, T50.2x3, T50.2x4, T50.3x1, T50.3x2, T50.3x3, T50.3x4, T50.4x1, T50.4x2, T50.4x3, T50.4x4, T50.5x1, T50.5x2, T50.5x3, T50.5x4, T50.6x1, T50.6x2, T50.6x3, T50.6x4, T50.7x1, T50.7x2, T50.7x3, T50.7x4, T50.8x1, T50.8x2, T50.8x3, T50.8x4, T50.901, T50.902, T50.903, T50.904, T50.911, T50.912, T50.913, T50.914, T50.991, T50.992, T50.993, T50.994, T50.A11, T50.A12, T50.A13, T50.A14, T50.A21, T50.A22, T50.A23, T50.A24, T50.A91, T50.A92, T50.A93, T50.A94, T50.B11, T50.B12, T50.B13, T50.B14, T50.B91, T50.B92, T50.B93, T50.B94, T50.Z11, T50.Z12, T50.Z13, T50.Z14, T50.Z91, T50.Z92, T50.Z93, T50.Z94, T51.0x1, T51.0x2, T51.0x3, T51.0x4, T51.1x1, T51.1x2, T51.1x3, T51.1x4, T51.2x1, T51.2x2, T51.2x3, T51.2x4, T51.3x1, T51.3x2, T51.3x3, T51.3x4, T51.8x1, T51.8x2, T51.8x3, T51.8x4, T51.91x, T51.92x, T51.93x, T51.94x, T52.0x1, T52.0x2, T52.0x3, T52.0x4, T52.1x1, T52.1x2, T52.1x3, T52.1x4, T52.2x1, T52.2x2, T52.2x3, T52.2x4, T52.3x1, T52.3x2, T52.3x3, T52.3x4, T52.4x1, T52.4x2, T52.4x3, T52.4x4, T52.8x1, T52.8x2, T52.8x3, T52.8x4, T52.91x, T52.92x, T52.93x, T52.94x, T53.0x1, T53.0x2, T53.0x3, T53.0x4, T53.1x1, T53.1x2, T53.1x3, T53.1x4, T53.2x1, T53.2x2, T53.2x3, T53.2x4, T53.3x1, T53.3x2, T53.3x3, T53.3x4, T53.4x1, T53.4x2, T53.4x3, T53.4x4, T53.5x1, T53.5x2, T53.5x3, T53.5x4, T53.6x1, T53.6x2, T53.6x3, T53.6x4, T53.7x1, T53.7x2, T53.7x3, T53.7x4, T53.91x, T53.92x, T53.93x, T53.94x, T54.0x1, T54.0x2, T54.0x3, T54.0x4, T55.0x1, T55.0x2, T55.0x3, T55.0x4, T55.1x1, T55.1x2, T55.1x3, T55.1x4, T56.0x1, T56.0x2, T56.0x3, T56.0x4, T56.1x1, T56.1x2, T56.1x3, T56.1x4, T56.2x1, T56.2x2, T56.2x3, T56.2x4, T56.3x1, T56.3x2, T56.3x3, T56.3x4, T56.4x1, T56.4x2, T56.4x3, T56.4x4, T56.5x1, T56.5x2, T56.5x3, T56.5x4, T56.6x1, T56.6x2, T56.6x3, T56.6x4, T56.7x1, T56.7x2, T56.7x3, T56.7x4, T56.811, T56.812, T56.813, T56.814, T56.891, T56.892, T56.893, T56.894, T56.91x, T56.92x, T56.93x, T56.94x, T57.0x1, T57.0x2, T57.0x3, T57.0x4, T57.1x1, T57.1x2, T57.1x3, T57.1x4, T57.2x1, T57.2x2, T57.2x3, T57.2x4, T57.3x1, T57.3x2, T57.3x3, T57.3x4, T57.8x1, T57.8x2, T57.8x3, T57.8x4, T57.91x, T57.92x, T57.93x, T57.94x, T58.01x, T58.02x, T58.03x, T58.04x, T58.11x, T58.12x, T58.13x, T58.14x, T58.2x1, T58.2x2, T58.2x3, T58.2x4, T58.8x1, T58.8x2, T58.8x3, T58.8x4, T58.91x, T58.92x, T58.93x, T58.94x, T59.0x1, T59.0x2, T59.0x3, T59.0x4, T59.1x1, T59.1x2, T59.1x3, T59.1x4, T59.2x1, T59.2x2, T59.2x3, T59.2x4, T59.3x1, T59.3x2, T59.3x3, T59.3x4, T59.4x1, T59.4x2, T59.4x3, T59.4x4, T59.5x1, T59.5x2, T59.5x3, T59.5x4, T59.6x1, T59.6x2, T59.6x3, T59.6x4, T59.7x1, T59.7x2, T59.7x3, T59.7x4, T59.811, T59.812, T59.813, T59.814, T59.891, T59.892, T59.893, T59.894, T59.91x, T59.92x, T59.93x, T59.94x, T60.0x1, T60.0x2, T60.0x3, T60.0x4, T60.1x1, T60.1x2, T60.1x3, T60.1x4, T60.2x1, T60.2x2, T60.2x3, T60.2x4, T60.3x1, T60.3x2, T60.3x3, T60.3x4, T60.4x1, T60.4x2, T60.4x3, T60.4x4, T60.8x1, T60.8x2, T60.8x3, T60.8x4, T60.91x, T60.92x, T60.93x, T60.94x, T61.01x, T61.02x, T61.03x, T61.04x, T61.11x, T61.12x, T61.13x, T61.14x, T61.771, T61.772, T61.773, T61.774, T61.781, T61.782, T61.783, T61.784, T61.8x1, T61.8x2, T61.8x3, T61.8x4, T61.91x, T61.92x, T61.93x, T61.94x, T62.0x1, T62.0x2, T62.0x3, T62.0x4, T62.1x1, T62.1x2, T62.1x3, T62.1x4, T62.2x1, T62.2x2, T62.2x3, T62.2x4, T62.8x1, T62.8x2, T62.8x3, T62.8x4, T62.91x, T62.92x, T62.93x, T62.94x, T64.01x, T64.02x, T64.03x, T64.04x, T64.81x, T64.82x, T64.83x, T64.84x, T65.0x1, T65.0x2, T65.0x3, T65.0x4, T65.1x1, T65.1x2, T65.1x3, T65.1x4, T65.211, T65.212, T65.213, T65.214, T65.221, T65.222, T65.223, T65.224, T65.291, T65.292, T65.293, T65.294, T65.3x1, T65.3x2, T65.3x3, T65.3x4, T65.4x1, T65.4x2, T65.4x3, T65.4x4, T65.5x1, T65.5x2, T65.5x3, T65.5x4, T65.6x1, T65.6x2, T65.6x3, T65.6x4, T65.811, T65.812, T65.813, T65.814, T65.831, T65.832, T65.833, T65.834, T65.891, T65.892, T65.893, T65.894, T65.91x, T65.92x, T65.93x, T65.94x, U07.0, Y35.201, Y35.202, Y35.203, Y35.209, Y35.211, Y35.212, Y35.213, Y35.219, Y35.291, Y35.292, Y35.293, Y35.299, Y36.7x0, Y36.7x1, Y37.7x0, Y37.7x1, Y38.7x1, Y38.7x2, Y38.7x3 |
| Struck by/against | V00.01x, V00.031x, V00.038x, V00.09x, V00.112, V00.122, V00.132, V00.142, V00.152, V00.182, V00.212, V00.222, V00.282, V00.312, V00.322, V00.382, V00.812, V00.822, V00.832, V00.842x, V00.892, V93.40x, V93.41x, V93.42x, V93.43x, V93.44x, V93.48x, V93.49x, W18.00x, W18.01x, W18.02x, W18.09x, W20.0xx, W20.1xx, W20.8xx, W21.00x, W21.01x, W21.02x, W21.03x, W21.04x, W21.05x, W21.06x, W21.07x, W21.09x, W21.11x, W21.12x, W21.13x, W21.19x, W21.210, W21.211, W21.220, W21.221, W21.31x, W21.32x, W21.39x, W21.4xx, W21.81x, W21.89x, W21.9xx, W22.01x, W22.02x, W22.03x, W22.041, W22.042, W22.09x, W22.10x, W22.11x, W22.12x, W22.19x, W22.8xx, W50.0xx, W50.1xx, W50.2xx, W50.3xx, W50.4xx, W51.xxx, W52.xxx, X79.xxx, Y00.xxx, Y04.0xx, Y04.1xx, Y04.2xx, Y04.8xx, Y08.01x, Y08.02x, Y08.09x, Y29.xxx, Y35.301, Y35.302, Y35.303, Y35.309, Y35.311, Y35.312, Y35.313, Y35.319, Y35.391, Y35.392, Y35.393, Y35.399, Y35.811, Y35.812, Y35.813, Y35.819, Y36.440, Y36.441, Y37.440, Y37.441 |
| Suffocation | T17.200, T17.210, T17.220, T17.290, T17.300, T17.310, T17.320, T17.390, T17.400, T17.410, T17.420, T17.490, T17.500, T17.510, T17.520, T17.590, T17.800, T17.810, T17.820, T17.890, T17.900, T17.910, T17.920, T17.990, T71.111, T71.112, T71.113, T71.114, T71.121, T71.122, T71.123, T71.124, T71.131, T71.132, T71.133, T71.134, T71.141, T71.143, T71.144, T71.151, T71.152, T71.153, T71.154, T71.161, T71.162, T71.163, T71.164, T71.191, T71.192, T71.193, T71.194, T71.20x, T71.21x, T71.221, T71.222, T71.223, T71.224, T71.231, T71.232, T71.233, T71.234, T71.29x, T71.9xx, Y36.460, Y36.461, Y36.470, Y36.471, Y37.460, Y37.461, Y37.470, Y37.471 |
| Unspecified | T14.91, X58.xxx, Y09., Y35.91x, Y35.92x, Y35.93x, Y35.99x, Y36.890, Y36.891, Y36.90x, Y37.90x, Y38.80x |
